# Targeting necroptosis in Alzheimer’s disease: can exercise modulate neuronal death?

**DOI:** 10.3389/fnagi.2025.1499871

**Published:** 2025-03-14

**Authors:** Donglei Lu, Wenyu Zhang, Ruiyu Li, Sijie Tan, Yan Zhang

**Affiliations:** ^1^Tianjin Key Laboratory of Sports and Health Integration and Health Promotion, Tianjin, China; ^2^First Teaching Hospital of Tianjin University of Traditional Chinese Medicine, Tianjin, China; ^3^Beijing University of Chinese Medicine Shenzhen Hospital (Longgang), Shenzhen, China; ^4^Tianjin Shengzhi Sports Technology Co., Ltd., Tianjin, China

**Keywords:** Alzheimer’s disease, necroptosis, exercise, neuronal degeneration, cognitive function

## Abstract

Alzheimer’s disease (AD) is a neurodegenerative disorder characterized by cognitive decline and neuronal degeneration. Emerging evidence implicates necroptosis in AD pathogenesis, driven by the RIPK1-RIPK3-MLKL pathway, which promotes neuronal damage, inflammation, and disease progression. Exercise, as a non-pharmacological intervention, can modulate key inflammatory mediators such as TNF-*α*, HMGB1, and IL-1β, thereby inhibiting necroptotic signaling. Additionally, exercise enhances O-GlcNAc glycosylation, preventing Tau hyperphosphorylation and stabilizing neuronal integrity. This review explores how exercise mitigates necroptosis and neuroinflammation, offering novel therapeutic perspectives for AD prevention and management.

## 1 Introduction

Alzheimer’s disease (AD) is a progressive, irreversible neurodegenerative disorder of the central nervous system, characterized by persistent cognitive impairment. The onset is gradual, with complex causative factors including genetic predispositions, spontaneous gene mutations, environmental influences, and infections (Zhengdong et al., 2022; Zhao-Tao et al., 2023). Hallmark pathological features of AD include extracellular amyloid-*β* (Aβ) deposits, intracellular neurofibrillary tangles (NFTs) of hyperphosphorylated tau protein, neuronal and synaptic loss, neurovascular modifications, and reactive gliosis (Scheltens et al., 2021; Askenazi et al., 2023). Despite extensive research, the precise mechanisms underlying neuronal death in AD remain poorly understood (Jack et al., 2024).

**Figure 1:**
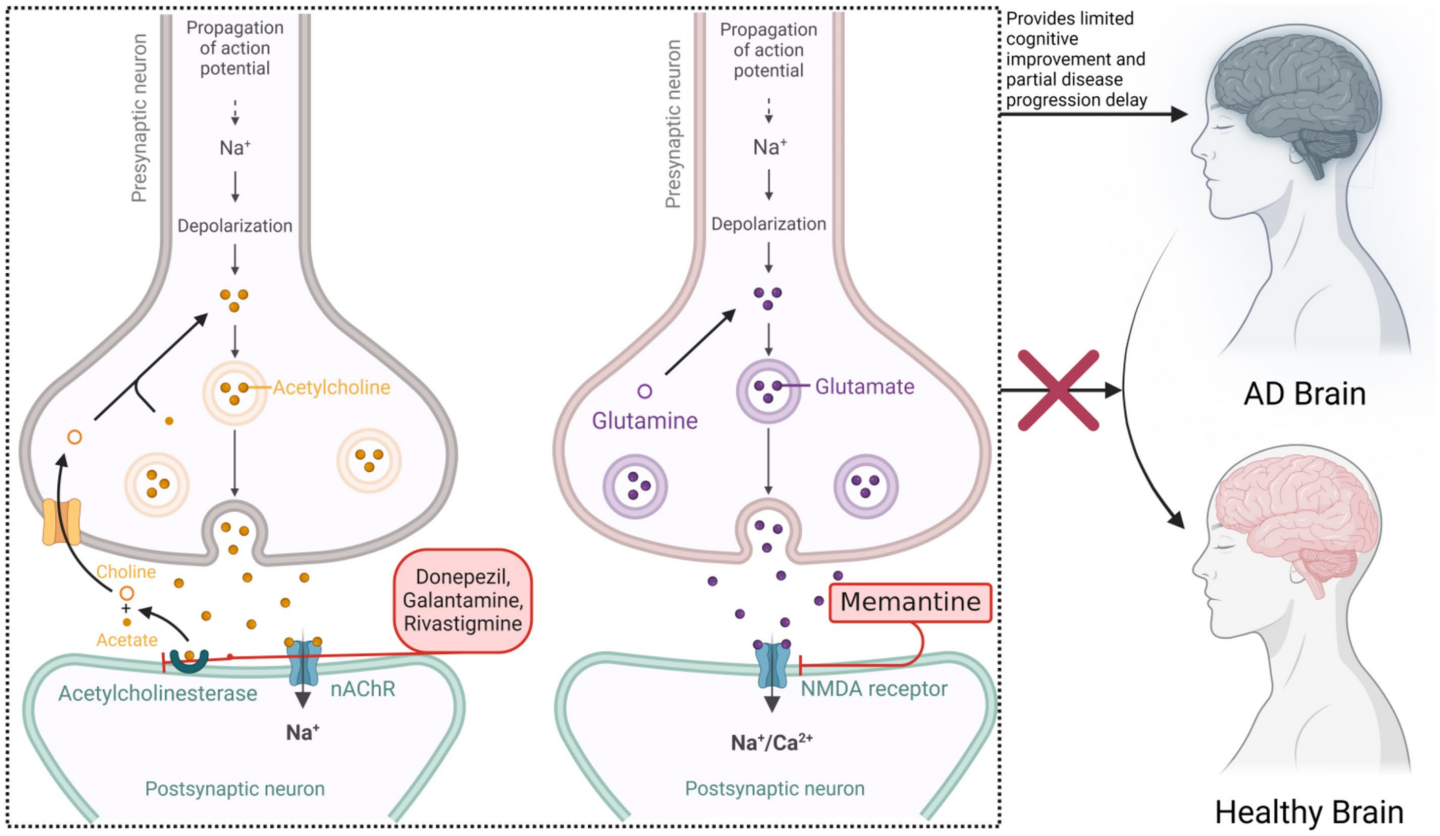
Mechanism of action of acetylcholinesterase inhibitors and NMDA receptor antagonists in Alzheimer’s disease.

Current pharmacological treatments, primarily acetylcholinesterase inhibitors (donepezil, galantamine, rivastigmine) and N-methyl-D-aspartate receptor antagonists (memantine), offer limited cognitive benefits and slow disease progression but do not halt or reverse the disease (Jaffee et al., 2024), see Figure 1. Recently, Aβ monoclonal antibody therapies such as aducanumab have shown promise in reducing amyloid burden; however, severe adverse effects like cerebral edema and microhemorrhages have significantly limited their clinical application (Cummings et al., 2024; Perneczky et al., 2024). Thus, balancing efficacy and safety remains a critical challenge in AD treatment.

Exercise therapy, known for its safety and minimal side effects, has increasingly gained attention among researchers and clinicians (Jun et al., 2023). Systematic exercise has been shown to enhance cognitive function, learning, and memory (Xu et al., 2023; Zhao, 2024). Nevertheless, while the beneficial effects of exercise on AD are acknowledged, the underlying biological mechanisms remain inadequately clarified (Baranowski et al., 2020). Emerging research suggests that exercise may mitigate AD through several mechanisms, including enhancing cerebral blood flow (Kaufman et al., 2021), improving mitochondrial function (Zhao et al., 2021), increasing neurotrophic factor levels (Wang X. et al., 2020), reducing oxidative stress (Wang W. et al., 2020), attenuating tau hyperphosphorylation (Wang et al., 2023a), and suppressing neuroinflammation (Quan et al., 2020). Pharmacological treatments for AD, such as acetylcholinesterase inhibitors and NMDA receptor antagonists, primarily aim to alleviate symptoms rather than modify disease progression. However, their limited long-term efficacy and the emergence of significant side effects underscore the need for alternative, adjunctive therapies. In this context, exercise offers a novel therapeutic avenue by targeting multiple disease mechanisms simultaneously. Unlike traditional pharmacological approaches, exercise not only improves cognitive function but also addresses underlying pathophysiological changes associated with AD, such as neuroinflammation and mitochondrial dysfunction, thereby presenting a complementary strategy with potential disease-modifying effects.

**Figure 2:**
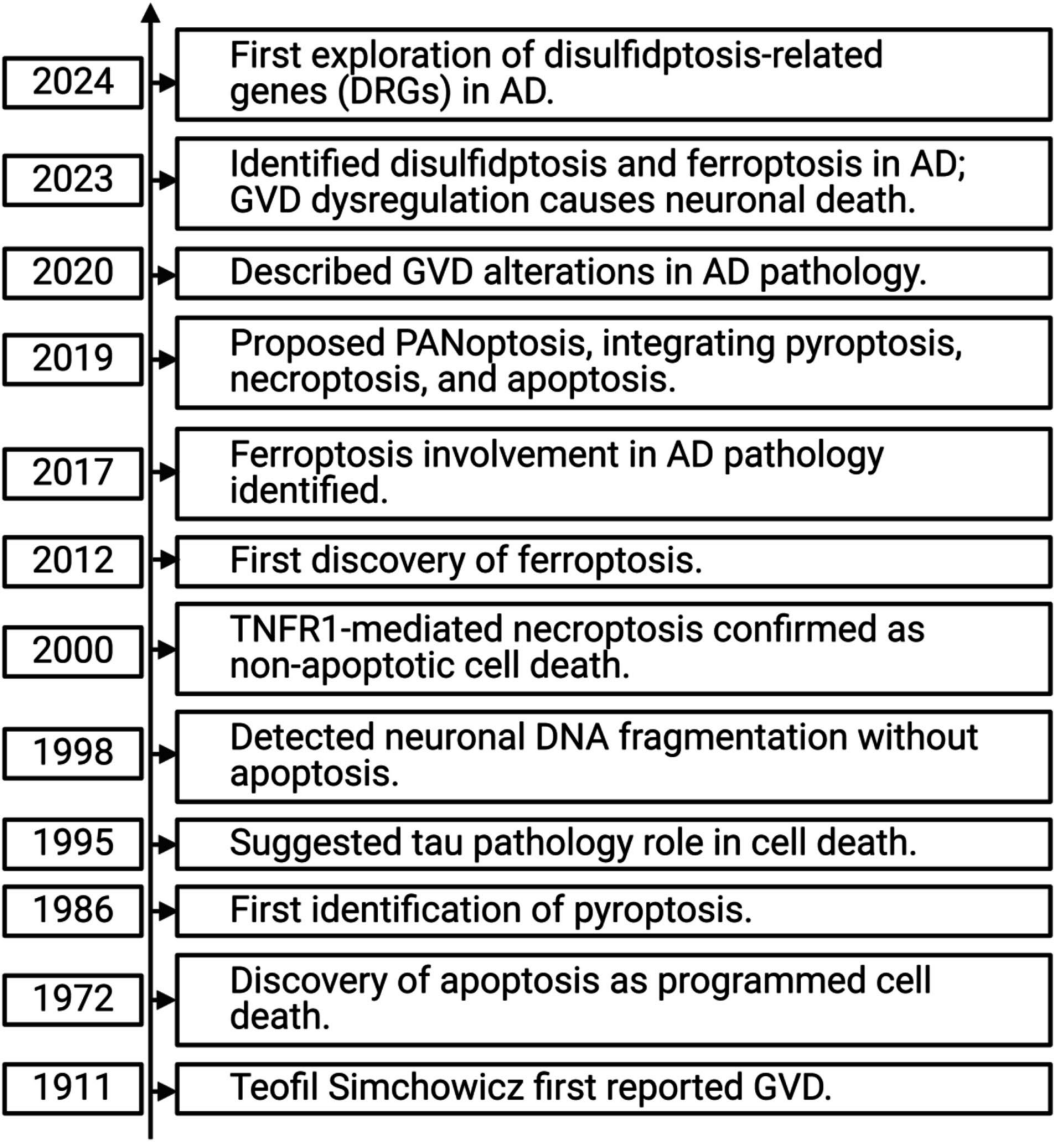
Timeline of cell death mechanisms in Alzheimer’s disease.

Recent studies have also highlighted the importance of various cell death modalities in AD pathology (see Figure 2), with necroptosis showing a strong correlation with AD onset and progression (Xu et al., 2021; Zhang et al., 2023). To date, no comprehensive study has explored the relationship between exercise, AD, and necroptosis. This review aims to: (1) investigate the role of necroptosis in AD pathogenesis, (2) examine the potential mechanisms linking necroptosis and AD progression, and (3) evaluate how exercise interventions may modulate necroptosis, offering insights into how exercise could be used as a therapeutic approach for AD. By addressing these objectives, this review seeks to provide a theoretical foundation for future exercise-based prevention and treatment strategies for AD, particularly in the context of necroptosis modulation.

## 2 Overview of necroptosis

**Figure 3:**
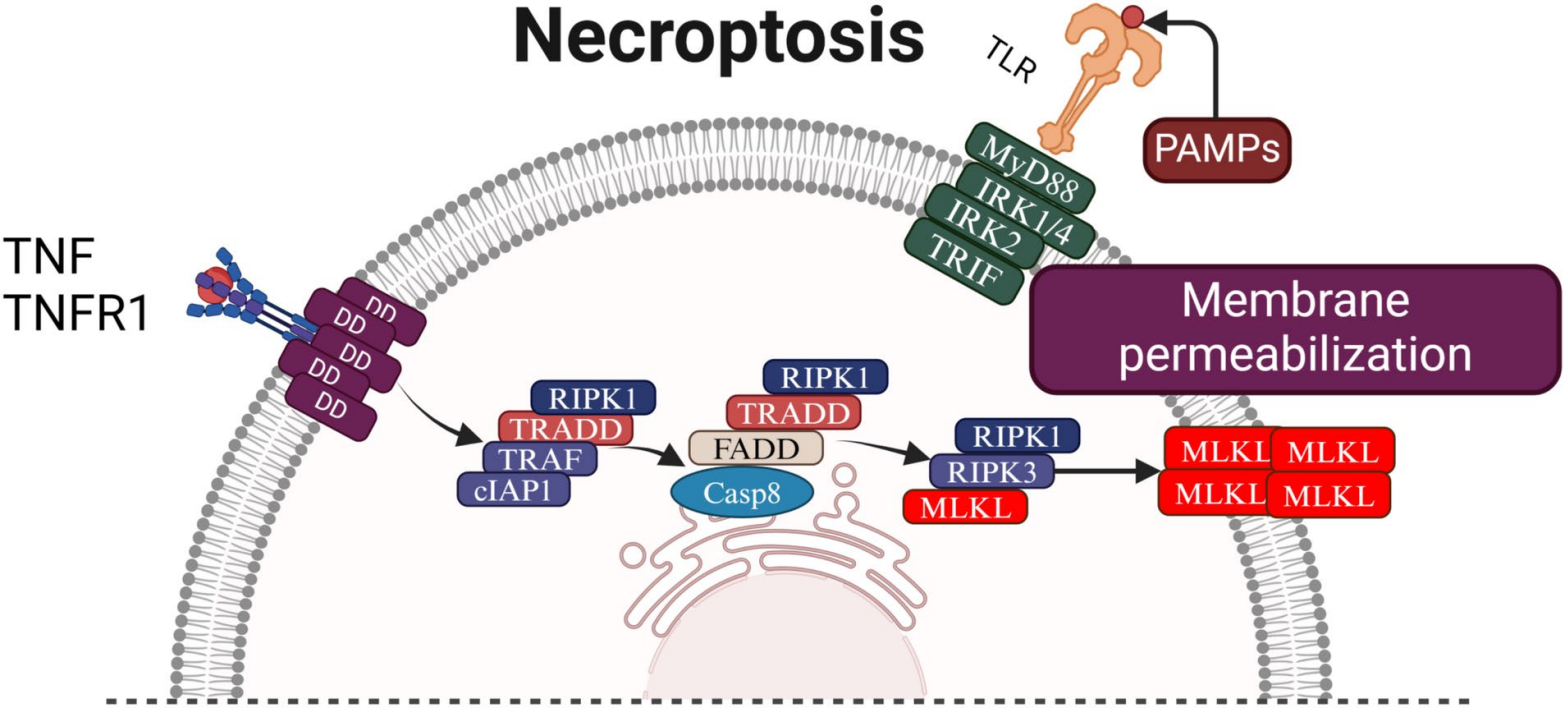
Mechanism of necroptosis: the figure delineates the mechanistic pathways of necroptosis initiation and execution. Necroptosis is primarily driven by TNF-α binding to TNFR1, which recruits RIPK1 to form Complex I. Upon deubiquitination, RIPK1 dissociates and, in the absence of active Caspase-8, assembles with RIPK3 into the necrosome, leading to MLKL phosphorylation and subsequent disruption of plasma membrane integrity. Additionally, interferon and TLR signaling pathways activate JAK/STAT and ISGF3, which further stimulate RIPK1/RIPK3-mediated necroptosis. Phosphorylated MLKL translocates to the plasma membrane, inducing Ca^2+^ influx via TRPM7 and releasing DAMPs that propagate inflammation. Mitochondrial ROS generation exacerbates cellular damage, establishing a feed-forward loop that intensifies necroptosis and inflammation, highlighting the interplay between cell death and immune responses. INF, Interferon; INFR, Interferon Receptor; JAKs, Janus Kinases; STAT1/2, Signal Transducer and Activator of Transcription 1/2; IRF9, Interferon Regulatory Factor 9; ISGF3, Interferon-Stimulated Gene Factor 3; PKR, Protein Kinase R; RIPK1, Receptor-Interacting Protein Kinase 1; RIPK3, Receptor-Interacting Protein Kinase 3; MLKL, Mixed Lineage Kinase Domain-Like Protein; TRADD, TNFR-Associated Death Domain; FADD, Fas-Associated Death Domain; Casp8, Caspase-8; cIAP1, Cellular Inhibitor of Apoptosis Protein 1; TRAF, TNF Receptor-Associated Factor; NF-κB, Nuclear Factor Kappa-Light-Chain-Enhancer of Activated B Cells; TNF, Tumor Necrosis Factor; TNFR1, Tumor Necrosis Factor Receptor 1; LPS, Lipopolysaccharide; TLR4, Toll-Like Receptor 4; MyD88, Myeloid Differentiation Primary Response 88; IRK1/4, Interleukin-1 Receptor-Associated Kinase 1/4; TRIF, TIR-Domain-Containing Adapter-Inducing Interferon-β; DNA, Deoxyribonucleic Acid; TRPM7, Transient Receptor Potential Melastatin 7; DAMPs, Damage-Associated Molecular Patterns.

The initiation, progression, regulation, and inhibition of necroptosis are complex processes involving numerous signaling molecules and proteins. Receptor-interacting serine/threonine-protein kinase 1 (RIPK1) and receptor-interacting serine/threonine-protein kinase 3 (RIPK3) play pivotal roles in modulating these processes. Concurrently, phosphorylation of mixed lineage kinase domain-like protein (MLKL) is the crucial link driving cellular membrane disruption (Hanson, 2016). Among the various necroptosis-inducing factors, the tumor necrosis factor-*α* (TNF-α)-mediated signaling pathway is the most extensively studied and well-characterized (see Figure 3).

## 3 Necroptosis and Alzheimer’s disease

### 3.1 Necroptosis and the primary pathological mechanism of Alzheimer’s disease

AD is defined by the aberrant accumulation of A*β* and tau proteins, which drive neurodegeneration. Aβ deposition begins decades before symptom onset, forming plaques that exacerbate neuronal dysfunction and cognitive decline (Caccamo et al., 2017). Aβ originates from amyloid precursor protein (APP) via sequential β-and *γ*-secretase cleavage. While APP processing can produce neuroprotective sAPPα, it also generates neurotoxic Aβ peptides, particularly oligomeric forms (Aβo), which disrupt synaptic function, promote neuronal loss, and activate inflammatory cascades (De la Rosa et al., 2020). Tau protein, a microtubule-associated protein predominantly expressed in neurons, stabilizes microtubules and supports axonal transport. Encoded on chromosome 17 (17q21), tau’s functional stability depends on its phosphorylation state. In AD, hyperphosphorylated tau aggregates into NFTs, destabilizing microtubules and impairing axonal function (Scheltens et al., 2021). Aβ and tau form a pathological feedback loop mediated by the PI3K/AKT/GSK-3β pathway. Aβ accumulation inhibits PI3K activity, relieving GSK-3β suppression, which promotes tau hyperphosphorylation. In turn, tau aggregation amplifies Aβ production by enhancing APP cleavage, accelerating plaque formation. This cycle is further compounded by chronic neuroinflammation. Neuroinflammation, driven by activated microglia and astrocytes, exacerbates neuronal injury (Trejo-Lopez et al., 2023). Aging and systemic inflammation disrupt blood–brain barrier integrity, allowing peripheral immune mediators to infiltrate the CNS, perpetuating a pro-inflammatory state. This chronic inflammation synergizes with Aβ and tau pathology, amplifying neuronal damage (Wang et al., 2023b). Recent evidence implicates necroptosis, a regulated form of necrotic cell death, as a downstream consequence of these processes. Unlike apoptosis, necroptosis exacerbates inflammation and tissue damage, representing a critical link between Aβ-tau interactions and AD progression.

**Figure 4:**
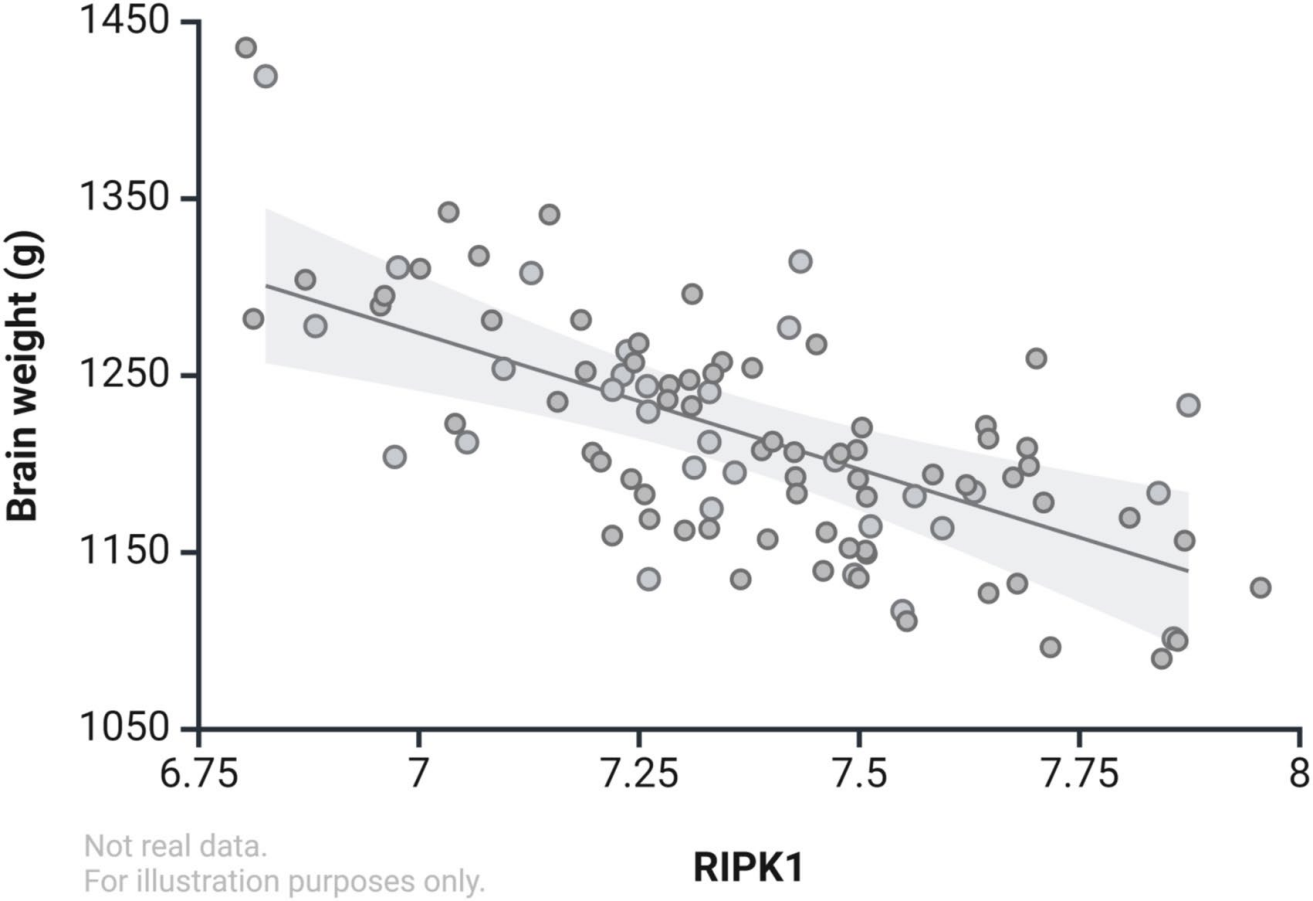
Correlation between RIPK1 levels and brain weight in Alzheimer’s disease (demonstrative data only, not actual values).

RIPK1 is a key mediator of necroptosis, contributing significantly to neuronal death in AD. RIPK1 expression is markedly elevated in advanced AD (higher Braak stages), correlating with reduced brain weight and suggesting a pivotal role in AD-associated neurodegeneration (Caccamo et al., 2017) see Figure 4. Necroptosis has been implicated as a key contributor to AD pathogenesis, with increased activation observed in both AD patients and animal models. Elevated levels of necroptosis-associated proteins, including RIPK1, RIPK3, and MLKL, have been consistently reported in sporadic AD brains. Notably, these proteins exhibit heightened colocalization, suggesting enhanced interaction in AD pathology. Upregulated mRNA expression of necroptosis markers further underscores their significance in AD progression. Phosphorylation of RIPK1 at S166, a marker of activation, is significantly increased in AD patient brains and AlCl3-induced AD rat models, alongside elevated RIPK3, phosphorylated MLKL, and CYLD levels, and decreased cIAP1 and cIAP2 expression. Necroptosis activation is also associated with blood–brain barrier (BBB) dysfunction, an early feature of AD characterized by loss of cerebral endothelial cells (ECs). In APP/PS1 transgenic mice and AD patients, RIPK1, RIPK3, and MLKL are significantly upregulated in ECs, while caspase-3 remains unchanged, indicating a necroptotic rather than apoptotic mechanism. RIPK1 activation in ECs contributes to BBB disruption and accelerates AD pathology. Granulovacuolar degeneration (GVD), another hallmark of AD, is linked to necroptosis, with phosphorylated RIPK1/3 and MLKL detected in GVD lesions during disease progression. Genetic predisposition may influence necroptosis in AD. A rare nonsynonymous SHARPIN variant impairs RIPK1 ubiquitination, enhancing RIPK1 activation and potentially elevating late-onset AD risk. Early white matter loss, observed prior to neurofibrillary tangles and amyloid plaques, also implicates necroptosis in initial AD pathology. Collectively, these findings establish necroptosis as a pivotal mechanism in AD, suggesting its potential as a therapeutic target. Interventions inhibiting key necroptosis molecules such as RIPK1, RIPK3, and MLKL may hold promise for mitigating AD-related pathology and cognitive decline. For more evidence, see Table 1.

**Table 1:** Evidence summary of necroptosis in Alzheimer’s disease.

Reference	Study type	Subjects	Methods/Intervention	Key findings	Conclusion
Xu et al. (2021)	Experimental, *in vitro*, in vivo, and clinical study	AD patients’ brain tissues, APP/PS1 and 5xFAD AD mouse models, SH-SY5Y, PC12, primary cortical neurons	IHC, immunoblotting, stereotaxic injection of TNF-α, AAV-mediated gene expression and knockdown, cell viability assays, PI staining, acridine orange staining	TNF-α/TNFR1 signaling initiates neuronal necroptosis via RIPK1/RIPK3/MLKL cascade. Impaired autophagy flux due to UVRAG downregulation leads to p62 accumulation, which exacerbates necroptosis in AD. TNFR1 knockdown or UVRAG overexpression reduces p-MLKL levels, inhibiting necroptosis.	TNF-α signaling modulates neuronal necroptosis in AD through the RIPK1/3 pathway and autophagy machinery. Targeting TNF-α/TNFR1 or UVRAG could be potential therapeutic strategies for AD.
Salvadores et al. (2022)	Experimental, in vitro, *in vivo* study	AD patients’ brain tissues, primary neurons, MLKL knockout mice	IHC, immunofluorescence, RNA extraction, stereotaxic injection of Aβ oligomers, behavioral assessment (Morris Water Maze), pharmacological inhibition of RIPK3	Aβ oligomers (Aβo) are associated with necroptosis activation (pMLKL) in the brains of AD patients. Aβo triggers microglial activation, leading to TNF-α release, which induces necroptosis in neurons through the TNFR1-RIPK1/RIPK3-MLKL pathway. Pharmacological inhibition of necroptosis prevents Aβo-induced neurodegeneration and memory loss.	Necroptosis is a key mechanism of neurodegeneration in AD, mediated by Aβo-induced microglial activation and TNF-α release. Targeting necroptosis offers potential therapeutic benefits for AD.
Park et al. (2021)	Experimental, in vitro, in vivo study	AD patients’ brain tissues, 5xFAD AD mouse models, primary neurons	IHC, immunofluorescence, RNA extraction, Western blot, stereotaxic injection of O-GlcNAc modulators, behavioral assessment (Morris Water Maze)	O-GlcNAcylation inhibits necroptosis in AD by reducing RIPK3 phosphorylation, thereby blocking its interaction with RIPK1. Enhanced O-GlcNAcylation prevents Aβ accumulation, neuronal loss, and neuroinflammation, and improves mitochondrial function and cognitive performance in AD mice.	O-GlcNAcylation modulates RIPK3 activity to suppress necroptosis and ameliorates AD-related pathological manifestations, making it a potential therapeutic target for AD treatment.
Caccamo et al. (2017)	Experimental, in vitro, in vivo study	AD patients’ brain tissues, APP/PS1 and 5xFAD AD mouse models, primary cortical neurons	IHC, immunoblotting, RNA extraction, stereotaxic injection of necroptosis inhibitors, behavioral assessment (Morris Water Maze)	RIPK1 and MLKL levels are elevated in human AD brains and correlate with Braak stage. Necroptosis is activated in AD neurons, indicated by the increase in MLKL phosphorylation and RIPK1/MLKL interactions. Pharmacological inhibition of necroptosis reduces neuronal loss and cognitive decline in AD mice.	Necroptosis contributes to neuronal death and cognitive deficits in AD. Targeting necroptosis could be a novel therapeutic approach for AD.
Koper et al. (2020)	Experimental, in vitro, in vivo, and clinical study	AD patients’ brain tissues, p-preAD and non-AD control cases, SH-SY5Y cells	IHC, immunofluorescence, Western blot, stereotaxic injection of AAV vectors, cell culture	Necrosome components (pRIPK1, pRIPK3, and pMLKL) are localized within granulovacuolar degeneration (GVD) lesions in AD brains, where they co-localize with classical GVD markers. The presence of necrosomes in GVD correlates with reduced neuronal density and severity of neurodegeneration.	GVD-related necroptosis may represent a specific mechanism of neuronal death in AD, offering potential for therapeutic intervention by targeting necrosome components in GVD.
Jayaraman et al. (2021)	Experimental, in vitro, in vivo, and clinical study	AD post-mortem brain tissues, human iPSC-derived glutamatergic neurons	IHC, Western blot, immunofluorescence, RT-qPCR, RNA extraction, pharmacological inhibition of necroptosis	TNF/TNFR1-mediated necroptosis pathway is significantly upregulated in AD hippocampal neurons, as shown by increased pRIPK3 and pMLKL levels. This upregulation correlates with increased neuron loss and is more pronounced in female AD brains. Pharmacological inhibition of necroptosis reduces TNF-induced neuronal death.	TNF signaling is a key mediator of necroptosis in AD neurons. Targeting TNF/TNFR1 signaling and the necroptosis pathway could be therapeutic strategies for preventing neuronal death in AD.
Balusu et al. (2023)	Experimental, in vitro, in vivo study	Human neurons xenografted into mouse AD models, human AD brain tissues	Xenotransplantation of human neurons into Rag2−/−/AppNL-G-F AD mouse model, RNA sequencing, immunohistochemistry, pharmacological inhibition of necroptosis	Human neurons in AD mouse models show upregulation of MEG3, a long noncoding RNA that induces necroptosis. MEG3 overexpression in human neurons leads to necroptosis through activation of RIPK1/RIPK3/MLKL pathway. Pharmacological or genetic inhibition of necroptosis rescues neuronal loss.	MEG3 is a critical mediator of necroptosis in human neurons and is upregulated in AD. Targeting MEG3 or downstream necroptosis pathways may offer therapeutic potential in AD treatment.

### 3.2 Necroptosis and apoptosis, neuronal death, and damage to the blood–brain barrier

The demise of hippocampal neurons is a defining pathogenic hallmark of AD closely linked to memory impairment. Neuronal loss in AD is not restricted to later stages; in specific brain regions, such as the entorhinal cortex layer II, Meynert basal nucleus, and locus coeruleus, degeneration occurs even before clinical symptoms emerge (Chu et al., 2022). This process involves two key cell death pathways: caspase-mediated apoptosis and death receptor-mediated necroptosis (Theofilas et al., 2018).

Apoptosis is characterized by cell shrinkage, membrane blebbing, and formation of apoptotic bodies, but caspase-dependent apoptosis is not the predominant mode of cell death in AD (Theofilas et al., 2022). Instead, neurons in AD often exhibit necroptotic features such as cellular swelling and DNA fragmentation, indicating that apoptosis alone cannot fully explain neuronal loss (Lassmann et al., 1995). Moreover, apoptosis typically manifests as a transient response, occurring over hours or days, while AD progression unfolds over decades (Kumari et al., 2023). Unlike apoptosis, which is “immune silent” and does not provoke an inflammatory response (Li and Yuan, 2008), necroptosis leads to cell membrane rupture, releasing intracellular contents and triggering an inflammatory cascade, potentially driving neuroinflammation in AD (Pasparakis and Vandenabeele, 2015). These findings suggest that neuronal death in AD involves more than apoptosis, with necroptosis playing a significant role.

Emerging evidence indicates that necroptosis is markedly activated in the brains of AD patients, characterized by increased expression of key necroptosis proteins RIPK1 and MLKL, leading to decreased neuronal survival and brain volume (Jayaraman et al., 2021). Activation of the TNFR1/RIPK1 signaling pathway and endosomal sorting complex required for transport III (ESCRT III) has also been observed in AD, implicating this route in the initiation of necroptosis (Jayaraman et al., 2021). ESCRT III is thought to have a cytoprotective role during late necroptosis, though its regulatory mechanisms require further study (Gong et al., 2017). Small molecule inhibitors targeting RIPK1, RIPK3, and MLKL have demonstrated a substantial reversal of necroptotic effects, indicating that neuronal death is an autonomous degenerative mechanism rather than solely a consequence of Aβ and Tau aggregation (Jayaraman et al., 2021).

In addition to necroptosis, other modes of programmed cell death, such as pyroptosis, ferroptosis, oxidative cell death, and autophagy, contribute to neuronal demise in AD. These pathways interact extensively, ultimately leading to neuronal loss, neuroinflammation, and exacerbated neurotoxicity.

The BBB is a key protective structure of the central nervous system, composed of endothelial cells, basement membranes, pericytes, and astrocytes. Its primary function is to maintain neurological homeostasis and protect the brain from harmful substances. Evidence suggests that BBB impairment is evident in the hippocampus of AD patients, potentially occurring in early stages of the disease (Kurz et al., 2022). Compromised BBB function not only accelerates Aβ accumulation but is also associated with genetic risk factors (notably the APOE ε4 allele) and cardiovascular conditions (including hypertension and diabetes) (Sweeney et al., 2015). In AD model mice and human samples, necroptotic cell death of endothelial cells appears to be the main driver of BBB breakdown and selective endothelial loss (Zou et al., 2020). Inhibition of RIPK1 kinase activity has been shown to enhance vascular permeability associated with TNF-*α* (Amin et al., 2018), and in acute brain injury models, Nec-1 inhibited BBB disruption in subarachnoid hemorrhage and cerebral ischemia/reperfusion injury (Fang et al., 2020).

Furthermore, a synergistic interaction between necroptosis and Aβ accumulation accelerates AD progression (Zhang et al., 2023). These observations suggest that necroptosis contributes significantly to BBB breakdown and the clinical progression of AD. Further research into the interplay between necroptosis and Aβ aggregation in BBB impairment may provide novel therapeutic opportunities for AD.

### 3.3 Necroptosis and neuroinflammation and mitochondrial dysfunction

Neuroinflammation is a prominent feature of AD, with microglia playing a central role in this process. As the primary immune cells of the brain, microglia work alongside astrocytes and oligodendrocytes to maintain central nervous system homeostasis and regulate neuroinflammatory responses (Lopez-Ortiz and Eyo, 2023). In AD, microglia exhibit two distinct phenotypes: the M1 pro-inflammatory phenotype, which exacerbates neuroinflammation and causes neuronal damage (Neniskyte et al., 2016), and the M2 anti-inflammatory phenotype, activated by IL-4 or IL-10, which aids in Aβ clearance (Hong et al., 2016). Previously, it was believed that Aβ plaques and neuronal degeneration triggered microglial activation, but genome-wide association studies have since shown that many AD risk genes are primarily expressed in microglia, suggesting a more complex role for microglia in AD pathology (Hemonnot et al., 2019).

Growing evidence indicates that necroptosis contributes not only to neuronal death but also to microglial activation. In retinal degeneration and acute retinal injury models, microglia promote neuroinflammation by releasing large amounts of pro-inflammatory cytokines and chemokines through RIPK1-and RIPK3-dependent necroptotic pathways (Huang et al., 2018). In an AD mouse model, elevated expression of necroptosis-related proteins, such as MLKL and Z-DNA/RNA-binding protein 1, was observed predominantly in M1 microglia, reinforcing the link between necroptosis and the pro-inflammatory M1 phenotype (Hao et al., 2021).

Moreover, the ablation of RIPK3 or MLKL facilitates the shift of microglia from an M1 to M2 phenotype, reducing neuroinflammation and enhancing Aβ clearance (Yang J. et al., 2018). In AD brain tissue, RIPK1 kinase activity is markedly increased, and its inhibition has been shown to reduce M1 microglia and pro-inflammatory marker expression (Wu et al., 2024). These findings underscore the pivotal role of necroptosis in mediating inflammatory responses and microglial dysfunction in AD, and suggest that targeting necroptosis could represent a promising therapeutic approach, particularly by promoting the M1-to-M2 microglial transition.

Mitochondrial dysfunction is another hallmark of AD. Mitochondrial respiratory chain complexes, especially complex IV, exhibit diminished activity in AD patients, leading to decreased ATP production, increased reactive oxygen species (ROS) levels, and DNA damage—factors that amplify Aβ accumulation and ultimately drive synaptic degeneration and cognitive decline (Wilkins and Swerdlow, 2021). Additionally, hyperphosphorylation of tau protein affects mitochondrial function and impairs axonal transport, contributing to neuronal dysfunction (Wang W. et al., 2020). Mitochondrial dysfunction is intricately linked to several aspects of AD pathology and has become a focal point of research.

Necroptosis and mitochondrial dysfunction are closely related. The mitochondrial phosphatase PGAM5 is an essential component of the RIPK1/RIPK3 complex. During necroptosis, the short isoform of PGAM5 (PGAM5S) mediates mitochondrial fragmentation by dephosphorylating dynamin-related protein 1 (Drp1), a critical event in necroptosis (Zhang et al., 2023). Necroptosis inhibitors that block the RIPK1/MLKL/PGAM5L axis can halt this process (Zhang et al., 2023). Moreover, necroptosis has been shown to drive active necrosome translocation to mitochondria through an MLKL-dependent pathway, enhancing aerobic respiration and ROS production via RIPK3 phosphorylation of the pyruvate dehydrogenase complex (Yang J. et al., 2018; Yang Z. et al., 2018). The resulting ROS further promote RIPK1 autophosphorylation, intensifying necroptosis (Pasparakis and Vandenabeele, 2015). These findings suggest that mitochondrial dysfunction not only contributes to necroptosis but also forms a detrimental feedback loop that accelerates AD progression. Inhibiting necroptosis may prevent mitochondrial damage and mitigate cognitive decline in AD patients.

### 3.4 Necroptosis and other pathological events

GVD, first identified in 1911, refers to membrane-bound structures measuring 3–5 μm, with a dense, silver-staining core surrounded by vacuoles (Köhler, 2016). In AD pathogenesis, GVD is frequently noted in hippocampal pyramidal neurons, especially within the CA1 and CA2 regions. As AD progresses, GVD lesions spread throughout cerebral regions, including the temporal lobe, hypothalamus, and amygdala (Koper et al., 2020). *In vitro* studies have demonstrated that intracellular Tau protein aggregation can induce GVD formation in primary mouse neurons (Wiersma et al., 2019). The number of GVDs in the hippocampus of AD patients increases with advancing NFT Braak stages, suggesting a link to neurofibrillary tangle formation (Hondius et al., 2021).

The Koper research group was the first to establish a correlation between GVDs and necroptosis in AD, indicating that GVDs are morphological markers of necroptosome activation in this context. Their findings revealed that regions with significant neuronal loss in pre-AD and AD brains exhibited numerous necrotic body-positive GVDs, implying that GVD-mediated necroptosis is a key mechanism underlying neuronal death (Koper et al., 2020). Additionally, TAR DNA-binding protein 43 (TDP-43) inclusions in the hippocampus have been strongly associated with GVD-mediated necroptosis. TDP-43 inclusions are found in up to 57% of AD cases and are linked to increased brain atrophy and memory impairment (Meneses et al., 2021). AD patients with TDP-43 exhibit a greater number of necrotic body-positive GVDs in the hippocampus compared to those without TDP-43 (Meneses et al., 2021). This phenomenon parallels findings in C9ORF72 mutation-related amyotrophic lateral sclerosis/frontotemporal dementia (Van Schoor et al., 2021). TDP-43 appears to exacerbate GVD-mediated necroptosis in both AD and ALS/FTLD, leading to more pronounced pathology and clinical manifestations.

**Figure 5:**
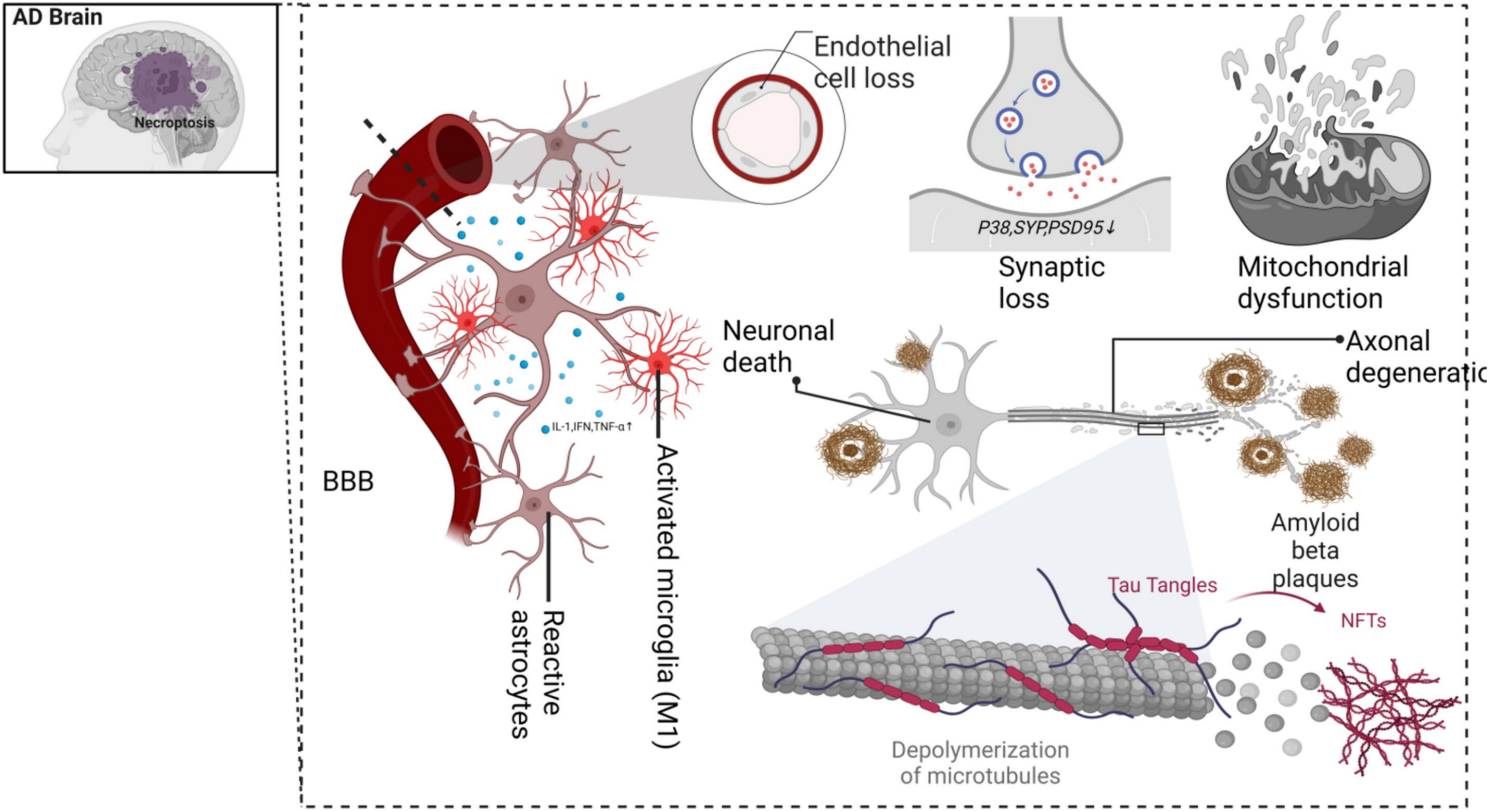
Alzheimer’s disease and necroptosis.

Moreover, axonal degeneration is a prominent feature in advanced AD, with necroptosis recognized as a primary mechanism by which excitotoxicity drives axonal loss (Hernández et al., 2018). Pharmacological inhibition of RIPK1 has been shown to block essential steps in axonal degeneration, such as mitochondrial depolarization, calcium dysregulation, and mitochondrial dysfunction within the axon (Arrázola et al., 2019), see Figure 5.

## 4 How does necroptosis impact AD cognition and neuropathology?

### 4.1 Role of necroptosis in cognition

A comprehensive review of previous studies suggests that necroptosis may impair cognitive function in AD through two primary pathways: reducing brain weight and decreasing neuronal count.

Caccamo et al. (2017) demonstrated an inverse correlation between brain weight and RIPK1 expression in AD patients after adjusting for height and weight. Furthermore, the Mini-Mental State Examination (MMSE), commonly used to assess cognitive function, showed a strong negative correlation between the expression levels of RIPK1 and MLKL and MMSE scores in AD patients, while no such correlation was observed in the control group. Notably, only the mRNA levels of MLKL and RIPK1, but not RIPK3, were significantly negatively correlated with MMSE scores in AD patients (Arevalo-Rodriguez et al., 2015). These findings suggest that activation of necroptosis may contribute to cognitive decline in AD by affecting brain weight. Consistent with this, the Morris water maze test indicated that necroptosis activation exacerbated cognitive decline in APP/PS1 transgenic mouse models (Zhao et al., 2022).

Koper et al. (2020) further reported elevated expression of pRIPK1, pRIPK3, and pMLKL in necrotic granules in the Gerstmann-Sträussler-Scheinker (GSS) lesion area, which was closely linked to significant neuronal loss in AD brain regions. In an AlCl3-induced AD rat model, Motawi et al. (2020) observed an increase in necrotic neurons in the CA1 region of the hippocampus, leading to reduced neuronal density. Similarly, Jayaraman et al. (2021) found a significant inverse correlation between total neuronal density and the cellular densities of pMLKL and pRIPK3 in AD patients, suggesting that necroptosis activation contributes to cognitive decline by reducing neuronal count. These findings highlight the critical role of necroptosis in neuronal damage and cognitive deterioration in AD, offering valuable insights for potential therapeutic targets.

### 4.2 Role of necroptosis in neuropathology

Neurofibrillary tangles represent a hallmark of AD pathology, and emerging evidence links their formation to necroptosis activation. Studies have demonstrated that mRNA levels of RIPK1 and MLKL are significantly elevated in AD patients, correlating positively with Braak staging, while RIPK3 levels show an inverse correlation (Braak and Braak, 1995). This suggests that RIPK1 and MLKL may play crucial roles in driving neurofibrillary tangle formation. Although no direct evidence currently supports RIPK1, RIPK3, or MLKL as predictors of *β*-amyloid plaque burden, Cst7—a disease-associated microglial (DAM) marker—has been shown to exhibit spatial proximity to β-amyloid plaques in both AD mouse models and postmortem human AD brain samples (Zhao et al., 2022). This association is mediated through a RIPK1-dependent mechanism. Further studies have revealed that necroptosis activation results in the formation of amyloid-like fibrils composed of RIPK1 and RIPK3 complexes (Li et al., 2012). These insoluble aggregates are markedly increased not only in AD patients but also in individuals with multiple sclerosis and amyotrophic lateral sclerosis, as well as in corresponding animal models (Ofengeim et al., 2015; Ito et al., 2016; Ofengeim et al., 2017). Experimental models have shown that RIPK1 inhibition or RIPK3 deficiency effectively reduces protein misfolding, indicating that these complexes may drive neurodegenerative processes by promoting pathological protein aggregation (Yuan et al., 2019).

Moreover, necroptosis triggers a robust neuroinflammatory response by activating microglia and upregulating pro-inflammatory mediators such as IL-1α, IL-1β, TNF, and IFNγ through a RIPK1-mediated, cell-autonomous pathway (Yuan et al., 2019). The accumulation of RIPK1-RIPK3 complexes also leads to NLRP3 inflammasome activation, exacerbating neuroinflammation via the suppression of RIPK1 inhibitors such as TAK1 and TBK1 (Lafont et al., 2018). This inflammatory cascade has been implicated in AD pathology, further linking necroptosis to disease progression. Importantly, RIPK1 has been shown to regulate the expression of CH25H, a key enzyme in lipid metabolism, and its downstream product 25-hydroxycholesterol, thereby affecting transcriptional profiles in the AD brain. Elevated CH25H expression has been observed in RIPK1-dependent pathways in AD mouse models and aging microglia, highlighting a novel connection between necroptosis and altered lipid metabolism in AD (Ofengeim et al., 2017; Mifflin et al., 2020).

In summary, RIPK1 exerts multifaceted effects in AD pathology, contributing to neurofibrillary tangle formation, β-amyloid plaque deposition, neuroinflammation, and transcriptional dysregulation. These findings position RIPK1 as a critical node in the disease’s complex network of pathological processes and suggest it as a potential therapeutic target for disrupting multiple deleterious pathways in AD.

## 5 Exercise and AD

### 5.1 The effect of exercise on AD

#### 5.1.1 Exercise training and tau phosphorylation and Aβ accumulation in the central nervous system

Exercise training exerts a profound effect on tau phosphorylation and Aβ accumulation in AD models. Long-term exercise interventions, such as a 5-month treadmill regimen, significantly mitigate Aβ deposition and tau hyperphosphorylation in the hippocampus of AD mice (Thomas, 2018). Mechanistically, exercise activates the glycogen synthase kinase 3 (GSK3) signaling pathway, which in turn reduces the phosphorylation of APP and attenuates the expression of presenilin 1, thereby inhibiting Aβ generation and tau hyperphosphorylation (Tan et al., 2021). Furthermore, exercise promotes the secretion of neurotrophic factors, which enhance hippocampal neuron survival and function, collectively decelerating the pathological progression of AD (Hu et al., 2024). Clinical studies corroborate these findings, indicating that exercise interventions positively influence Aβ accumulation and tau phosphorylation in human subjects with AD.

#### 5.1.2 Exercise training and brain-derived neurotrophic factor (BDNF) expression in the central nervous system

Exercise training has been consistently shown to upregulate the expression of BDNF, thereby promoting neurogenesis and synaptic plasticity. In animal models of AD, exercise increases BDNF levels, thereby enhancing hippocampal neuroplasticity and facilitating synaptic function recovery (Luo et al., 2019). Aerobic exercise, for instance, notably elevates BDNF expression in the hippocampus of AD mice, and this increase correlates closely with cognitive improvement (Luo et al., 2019). Moreover, BDNF exerts its neuroprotective effects by binding to its receptor TrkB, activating downstream signaling pathways that promote long-term memory consolidation and dendritic remodeling (Soman et al., 2024). Clinical research further substantiates the role of exercise in augmenting BDNF expression in AD patients. For example, six months of continuous aerobic exercise significantly elevates plasma BDNF levels in AD patients (Enette et al., 2020), with a positive correlation to cognitive enhancement. These findings suggest that BDNF may serve as a crucial mechanism through which exercise exerts its cognitive benefits in AD.

#### 5.1.3 Exercise training and inflammation in the central nervous system

Neuroinflammation is a hallmark of Alzheimer’s disease, typified by the activation of microglia and astrocytes and the subsequent release of pro-inflammatory cytokines. Exercise training has emerged as a potent modulator of neuroinflammatory responses, effectively reducing levels of inflammatory markers such as TNF-*α*, IL-6, and COX-2 in the brain. In animal models of AD, regular exercise significantly diminishes inflammation in the hippocampus and other brain regions, concomitantly reducing Aβ deposition and tau phosphorylation (Nakanishi et al., 2021). For instance, after 4 months of moderate-to-high intensity exercise, AD mice exhibit a marked reduction in inflammatory markers and Aβ accumulation in the hippocampus, coupled with improved cognitive performance (Nakanishi et al., 2021). Additionally, exercise fosters the secretion of anti-inflammatory cytokines such as IL-10 and IL-1RA, further attenuating neuroinflammation in AD mice (Batista et al., 2010). Clinical studies also demonstrate that exercise can modulate neuroinflammatory responses in AD patients. For example, a 12-week aerobic exercise program significantly reduces plasma TNF-*α* and IL-6 levels while increasing IL-10 concentrations, suggesting that exercise may improve cognitive function by modulating neuroinflammation (Robinson et al., 2018).

#### 5.1.4 Exercise training and oxidative stress in the central nervous system

Oxidative stress plays a pivotal role in the pathophysiology of Alzheimer’s disease. Exercise training has been shown to mitigate oxidative stress by enhancing mitochondrial function, thereby slowing the progression of AD (Bo et al., 2014). Studies indicate that exercise improves mitochondrial morphology, enhances mitochondrial autophagy, and reduces Aβ deposition, collectively alleviating cognitive deficits in AD rodents (Liang et al., 2021). High-intensity interval training (HIIT) and moderate-intensity continuous exercise, for example, significantly reduce Aβ accumulation in the hippocampus, enhance mitochondrial structure, and suppress oxidative stress, leading to improved spatial learning and memory (Liang et al., 2020). Additionally, exercise increases NAD^+^ levels, which in turn promotes mitochondrial autophagy and alleviates mitochondrial dysfunction associated with AD (Zhao et al., 2023). Clinical research further supports these findings, showing that aerobic exercise over 16 weeks significantly reduces oxidative stress markers such as MDA and SOD in the serum of AD patients, with concurrent cognitive improvements (Delgado-Peraza et al., 2023). These studies suggest that exercise may serve as a critical intervention for modulating oxidative stress, improving mitochondrial health, and ameliorating the pathological features of AD (see in Tables 2, 3).

**Table 2:** Effects of physical exercise on brain metabolism in animal models.

Study (Author and year)	Rats, Model, Sex	Experimental groups	Exercise intervention	Changes in outcome measures
Khodadadi et al. (2018)	Male Wistar rats, 8 weeks old, Alzheimer’s disease model (hippocampal injection of Aβ1–42)	Control group, Exercise group, Aβ group, Aβ + Exercise group, Sham group	Treadmill exercise (moderate intensity, 4 weeks, 5 days/week)	Treadmill exercise significantly improved learning and memory, reduced Aβ plaques and sAβ1–42 in the brain and blood, and upregulated the expression of NEP, IDE, and LRP-1 in a rat model of Alzheimer’s disease, with statistically significant differences (*p* < 0.05 to *p* < 0.001).
Choi et al. (2021)	24-month-old APP-C105 Alzheimer’s transgenic mice	NTg-C (Non-transgenic control group), NTg-TE (Non-transgenic exercise group), Tg-C (Transgenic control group), Tg-TE (Transgenic exercise group)	Treadmill exercise (moderate intensity, 8 weeks, 5 days/week)	Treadmill exercise enhanced cognitive function in APP – C105 mice, shown by improved performance in multiple tests with significant differences (Morris water maze, passive avoidance, novel object recognition; *p* < 0.001 or *p* = 0.001). It reduced motor cortex iron (Fe^2+^, Fe^3+^, total Fe; *p* = 0.001), increased antioxidant enzyme activity (SOD1 etc.; *p* = 0.001 or 0.014), decreased Aβ₄₂ (correlated with iron; *p* < 0.001), and attenuated neuronal death (*p* = 0.001).
Pang et al. (2019)	6-month-old male APP/PS1 double transgenic mice, Alzheimer’s disease model	WT-NT (Wild-type control group), WT-T (Wild-type exercise group), AD-NT (Alzheimer’s control group), AD-T (Alzheimer’s exercise group)	Swimming training (1 h/day, 6 days/week, for 4 weeks)	Regular swimming exercise (6 days per week, 1 h per day, for 4 weeks) significantly improved cognitive function in Alzheimer’s disease (AD) model mice (*p* < 0.05), reduced Aβ and P-Tau expression (*p* < 0.01), increased synaptic density (*p* < 0.01) and ATP levels (*p* < 0.01), and enhanced GLUT1 and GLUT3 expression (*p* < 0.05), alleviating AD pathology and cognitive impairment by improving energy metabolism and neuroplasticity.
Zhang et al. (2022)	10-month-old male APP/PS1 double transgenic mice	AD_Sed (Alzheimer’s sedentary group), AD_Run (Alzheimer’s exercise group), WT_Sed (Wild-type sedentary group), WT_Run (Wild-type exercise group)	Voluntary wheel running (12 cm diameter wheel, free activity, for 3 months)	Long-term running exercise (3 months, voluntary wheel running) significantly improved cognitive function in APP/PS1 mice (*p* < 0.05), increased hippocampal glucose metabolism (*p* < 0.01) and microglial GLUT5 expression (*p* < 0.05), enhanced microglial morphological plasticity (*p* < 0.05), maintained TREM2 protein levels, and reduced sTREM2 release (*p* < 0.05), alleviating AD-related pathology and cognitive impairment by promoting glucose metabolism.
Li et al. (2022)	8-week-old male ICR rats, Alzheimer’s disease model (hippocampal injection of Aβ1-42)	Saline group, Aβ group, Aβ + Exercise group, Aβ + Exercise+40 Hz stimulation group	Treadmill exercise (passive, 4 weeks) + voluntary wheel running (voluntary, 4 weeks) + light and sound stimulation (40 Hz, 4 weeks)	Passive and voluntary exercise combined with 40 Hz acousto-optic stimulation significantly increased BrdU^+^ newborn cells and DCX^+^ immature neurons in the hippocampus of AD mice (*p* < 0.001), promoting differentiation into NeuN^+^ mature neurons and GFAP^+^ astrocytes (*p* < 0.0001). The intervention improved cognitive function (*p* < 0.05), reduced anxiety-and depression-like behaviors (*p* < 0.01), activated the BDNF/TrkB/Akt pathway, and regulated metabolic pathways.
Sarlak et al. (2019)	8-week-old male Wistar rats, Alzheimer’s disease model (hippocampal injection of Aβ1-42)	Control group, Exercise group, Aβ group, Aβ + Exercise group, Sham group	Treadmill exercise (moderate intensity, 4 weeks, 5 days/week)	Aerobic training significantly increased ABCA1 mRNA expression (*p* < 0.05) and reduced soluble Aβ1-42 levels in the hippocampus (*p* < 0.01) of AD-induced rats, with the greatest effects observed in pre-and post-conditioning groups. Cognitive function improved significantly, as shown by decreased escape latency and swimming distance (*p* < 0.01) and increased time in the target quadrant (*p* < 0.01).
Yuanyuan (2024)	Male C57BL/6 mice, type 2 diabetes mellitus model (STZ induction)	Control group (CON), T2DM model group, T2DM + HIIT group	High-intensity interval training (HIIT, 7 weeks, 6 days/week, 60 min/session)	High-intensity interval training (HIIT) significantly reduced hippocampal necroptosis in T2DM mice by decreasing LDH release (*p* < 0.0001), PI-positive cells (*p* < 0.05), and RIPK1/RIPK3/MLKL protein levels (*p* < 0.05). HIIT also suppressed NLRP3 inflammasome activation and pro-inflammatory cytokines TNF-α, IL-1β, IL-6, and iNOS expression (*p* < 0.05), effectively mitigating inflammation and protecting hippocampal neurons.
Zeini Zadeh et al. (2023)	4-6-week-old male Wistar rats, Alzheimer’s disease model (STZ induction)	C-H (Healthy control group), C-ALZ (Alzheimer’s control group), CWheel-ALZ (Complex training group)	Complex training (12 weeks, voluntary wheel running)	Complex voluntary training significantly reduced hippocampal necroptosis-related gene expression, including RIPK1, MLKL, and TNFR1 (*p* < 0.05), increased CA1 pyramidal cell layer thickness and neuronal density (*p* < 0.01), and improved spatial memory in AD rats (*p* < 0.05). However, deficits remained compared to healthy controls, indicating partial but not complete reversal of AD-induced impairments.
Leem et al. (2024)	7-week-old male C57BL/6 mice, Parkinson’s disease model (MPTP induction)	CON (Healthy control group), M/P (Parkinson’s model group), M/P + Ex (Parkinson’s + Exercise group), M/P + Cr (Parkinson’s + Creatine group), M/P + Ex+Cr (Combined group)	Creatine supplementation (2%) + rotarod exercise (4 weeks, 5 days/week)	Creatine supplementation combined with exercise significantly improved motor function (*p* < 0.01) and increased tyrosine hydroxylase levels in Parkinson’s disease mice. It reduced necroptosis markers (MLKL, RIPK1/RIPK3) and α-synuclein aggregation (*p* < 0.01), suppressed inflammation (*p* < 0.01), and enhanced antioxidant enzyme levels via AMPK/Nrf2 and SIRT3/FoxO3a pathways (*p* < 0.01), showing additive neuroprotective effects.

**Table 3:** Efects of physical exercise on brain metabolism in human.

Study (Author and year)	Participants	Experimental groups	Exercise intervention	Changes in outcome measures
Gaitán et al. (2019)	23 late-middle-aged adults with familial and genetic risk for AD, mixed-sex	Usual Physical Activity (PA) group, Enhanced PA group	26 weeks of supervised aerobic treadmill exercise, 3 sessions/week, 50 min/session at 70–80% heart rate reserve	After 26 weeks of aerobic exercise, the Enhanced Physical Activity group showed a 3.89 mL/kg/min improvement in cardiorespiratory fitness (*p* = 0.018), 27.36 min/day less sedentary time (*p* = 0.003), and 28.47 min/day more moderate-to-vigorous activity (*p* = 0.008). Executive function improved significantly by 7.18 s (*p* = 0.022), correlating with fitness gains.
Robinson et al. (2018)	27 participants (18–30 years and 65–80 years old), mixed-sex	High-intensity interval training (HIIT) group, Sedentary (SED) control group	12 weeks of HIIT (3 sessions/week of 4×4 minute intervals at over 90% peak aerobic capacity (VO2peak) + 2 sessions/week of treadmill walking at 70% VO2peak)	After 12 weeks of HIIT, brain glucose uptake significantly increased in the left temporal lobe (+0.358, *p* = 0.029) and right caudate nucleus (+0.473, *p* = 0.012) compared to the sedentary group. VO2peak significantly improved in the HIIT group (*p* < 0.001), while no changes occurred in the sedentary group. Improvements in brain glucose uptake were region-specific and not observed globally across all brain areas.
Delgado-Peraza et al. (2023)	95 patients with mild to moderate AD, APOE ε4 carriers, mixed-sex	Exercise group, Control group	16 weeks of moderate to high-intensity aerobic exercise	After 16 weeks of exercise, proBDNF in NDEVs increased 1.8-fold (*p* = 0.007), while humanin increased significantly (*p* = 0.018). Both biomarkers showed more prominent increases in APOE ε4 carriers. BDNF levels also increased but did not reach significance. No changes occurred in the control group, and exerkines remained unchanged, suggesting neuron-specific effects of exercise.
Grzenda et al. (2024)	79 postmenopausal women with subjective cognitive decline (SCD) and cardiovascular risk factors	Kundalini yoga (KY) group, Memory enhancement training (MET) group	12 weeks of weekly 60-min in-person KY sessions + daily 12-min guided practice; 12 weekly in-person MET sessions +12-min daily homework	At 24 weeks, Kundalini Yoga (KY) significantly improved the seriousness of forgetting score by +0.65 (*p* = 0.04), while the Memory Enhancement Training (MET) group showed no significant change (−0.31, *p* = 0.4). KY participants demonstrated a decline in delayed recall (−0.31, *p* = 0.0003) compared to MET (+0.02, *p* = 0.6), with a significant group difference (*p* = 0.002). No significant differences were observed in executive functioning.

### 5.2 Potential mechanisms of necroptosis-mediated exercise prevention and treatment of AD

#### 5.2.1 Potential mechanisms of exercise-mediated treatment of AD through upregulation of irisin in necroptosis

Irisin was first identified by Bostrom in 2012 as a “myokine” synthesized by skeletal muscle. It is released through the cleavage of Fibronectin Type III Domain-Containing Protein 5 (FNDC5), a process stimulated by exercise-induced PGC1-*α* expression. Not only is irisin present in skeletal and cardiac muscles, but it is also widely distributed in organs like the brain, liver, and pancreas (Boström et al., 2012). This hormone plays a pivotal role in metabolic regulation, as evidenced by its effects on browning white adipose tissue, enhancing glucose metabolism, and reducing inflammatory responses—particularly in AD (Lourenco et al., 2019b). Irisin can cross the blood–brain barrier, promote neurotrophic factor expression in the hippocampus, stimulate neurogenesis, protect neurons from damage, and improve cognitive and memory functions (Guo et al., 2021).

Recent findings suggest that Irisin may alleviate arthritis through suppression of the necroptosis pathway, implying that it might also regulate inflammation and apoptotic signaling—positioning it as a potential therapeutic target for neurological disorders like AD (Raafat et al., 2022). Irisins might exert neuroprotective effects in AD by modulating several molecular mechanisms, including the suppression of necroptosis via pathways involving TNF-*α* signaling, HMGB1/MCP1-mediated inflammation, and BDNF regulation.

The TNF-α signaling pathway is a critical route for neuronal necroptosis activation. In AD, TNF-α levels are significantly elevated, activating the RIPK1-RIPK3-MLKL pathway, which leads to cell membrane damage, neuronal necroptosis, and intensified neuroinflammation (Kim et al., 2025). Irisin glycosides are shown to attenuate this cascade by inhibiting TNF-*α* mRNA expression, thereby protecting neurons from necroptotic injury (Kim et al., 2025). The anti-inflammatory effects of Irisins have been demonstrated in both *in vitro* and *in vivo* studies, with mechanisms involving multiple signaling pathways. For instance, in hippocampal astrocytes, Irisin reduced inflammation by inhibiting the NF-κB/IκBα pathway, subsequently lowering IL-1β and IL-6 levels (Heurtaux et al., 2022). In ischemia–reperfusion injury models, Irisin glycosides reduced inflammation and neuronal damage by suppressing the TLR4/MyD88/NF-κB pathway (Yu et al., 2020).

Studies have also demonstrated the capacity of Irisin glycosides to inhibit hippocampal apoptosis and reduce pro-inflammatory cytokines IL-1β and TNF-α (Bellettini-Santos et al., 2023). In an animal model, intravenous administration of Irisin suppressed microglial activation and decreased the production of IL-6 and TNF-α through the AKT and ERK1/2 pathways, thus mitigating ischemia-induced neuronal damage (Li et al., 2017). Furthermore, high-dose Irisin administered intraventricularly was found to significantly increase UCP5 mRNA expression in regions such as the hippocampus, striatum, and hypothalamus, leading to a decrease in neuroinflammation (Erden et al., 2016). In patients with central nervous system inflammation, exercise—both single and prolonged cycling—elevated irisin levels while reducing IL-6 (Briken et al., 2016). Liu et al. (2020) observed that short-term resistance training in a 3xTg AD mouse model increased irisin expression, reduced microglial activation in the hippocampus, and decreased levels of TNF-α and IL-1β mRNA. Concurrently, anti-inflammatory IL-10 expression increased. These effects indicate that FNDC5/irisin signaling, activated during exercise, enhances neuroinflammatory responses by modulating microglial activity and potentially inhibiting TNF-α-induced necroptosis (Kim et al., 2025).

Irisins are also believed to counter inflammation-induced neuronal injury by suppressing HMGB1 and Monocyte Chemoattractant Protein-1 (MCP-1), thereby blocking the activation of RIPK1 and RIPK3 signaling by inflammatory mediators (Raafat et al., 2022). HMGB1, a key pro-inflammatory mediator, intensifies inflammation by activating immune cells and promoting necroptosis (Bian et al., 2023), while MCP-1 recruits immune cells, exacerbating local neuroinflammation (Ghosh et al., 2021). Irisins significantly lower HMGB1 and MCP-1 expression, mitigate the inflammatory cascade, and may help manage chronic brain inflammation in AD (Mazur-Bialy, 2019). In a study involving RAW 264.7 macrophages, Irisin glycosides suppressed the TLR4/MyD88 signaling pathway, reducing LPS-induced inflammation and the secretion of pro-inflammatory factors including TNF-*α*, IL-1β, IL-6, MCP-1, and HMGB1 (Mazur-Bialy et al., 2017). In the context of AD, inhibition of the TLR4/MyD88 pathway by Irisins could reduce HMGB1 and MCP-1 expression, effectively mitigating inflammation-induced neuronal damage and slowing disease progression (Ruganzu et al., 2022).

Irisin compounds can also enhance neuronal survival and repair by upregulating brain-derived neurotrophic factor (BDNF) expression (Tagai et al., 2020). BDNF is thought to exert a dual effect on AD pathogenesis, with altered levels influencing neuronal survival and synaptic plasticity (Banerjee and Shenoy, 2023). Research using SH-SY5Y cells found that diminished BDNF impeded axonal growth and led to neuronal fragmentation in response to A*β* peptides, which is primarily driven by caspase-6-dependent necroptosis (Krishtal et al., 2017). Necroptosis inhibitors reduced Aβ-induced neuronal death, highlighting its role in cell death linked to reduced BDNF and Aβ aggregation (Krishtal et al., 2017). Conversely, BDNF overexpression promotes neuronal proliferation via the PI3K/Akt and MAPK/ERK pathways while reducing oxidative stress damage (Tagai et al., 2020). BDNF also inhibits necroptosis by blocking RIPK1 and MLKL production through PI3K/Akt signaling (Li et al., 2021). Exercise, a key modulator of BDNF levels, activates the PGC-1α/Irisin/BDNF pathway, helping to protect neurons from ROS damage and alleviate cognitive decline (Lourenco et al., 2019a). In a study on obese mice, exercise improved PGC-1α, irisin, and BDNF expression in the prefrontal cortex and reduced inflammation and oxidative stress, improving cognition (Peng, 2023). High-intensity interval training was particularly effective in this regard. Zeini Zadeh et al. (2023) showed that exercise in an AD rat model reduced neuroinflammation and improved spatial memory by increasing hippocampal BDNF and reducing RIPK1, MLKL, and TNFR1 expression. These findings underscore the role of BDNF in promoting neuronal survival, synaptic plasticity, and functional recovery in AD.

#### 5.2.2 Neuroprotective mechanism of RIPK1-RIPK3-MLKL necroptosis pathway in Alzheimer’s disease

In AD models, the expression levels of RIPK1, RIPK3, and MLKL are significantly elevated, indicating active necroptosis (Zeini Zadeh et al., 2023). Experimental evidence shows that in the hippocampus of AD model rats, RIPK1 expression increased 1.79-fold, MLKL increased 2.19-fold, and TNFR1 increased 2.81-fold, leading to activation of the RIPK1-RIPK3-MLKL pathway. This activation results in extensive necroptosis of hippocampal neurons and a strong inflammatory response. Necroptosis further exacerbates neuroinflammation by releasing pro-inflammatory molecules, establishing a vicious cycle of inflammation and neuronal death that drives AD progression.

Exercise intervention has shown regulatory effects on this pathway. Zeini Zadeh et al. (2023) demonstrated that after 12 weeks of intensive exercise, RIPK1 expression in AD model rats was reduced to 1.44 times, MLKL to 1.66 times, and TNFR1 from 2.81 to 1.64 times. These findings suggest that exercise can suppress RIPK1 activation by modulating the TNF-*α*/TNFR1 pathway, thereby reducing necroptosis. This suppression diminishes RIPK3 phosphorylation, inhibits MLKL activation, and mitigates its harmful effects on neuronal membranes. By blocking the RIPK1-RIPK3-MLKL pathway, exercise can significantly decrease neuronal death, enhance neuronal survival in the hippocampus, and slow the molecular course of AD. Furthermore, exercise reduces oxidative stress by decreasing reactive oxygen species (ROS) generation, which is linked to β-amyloid accumulation—a driver of inflammation and necroptosis in AD. Thus, exercise offers a therapeutic strategy to mitigate AD progression by reducing ROS and breaking the cycle of neurodegeneration.

Behavioral assessments support the neuroprotective effects of exercise. The Morris water maze test showed that exercise training significantly reduced escape latency in AD model rats, indicating improved spatial memory. Histological analysis revealed increased neuronal density in the hippocampal CA1 region of the exercise group—from 67.7/μm^2^ in controls to 84.95/μm^2^—approaching the healthy control level of 100.88/μm^2^. This highlights the role of exercise in promoting neuronal survival and cognitive function.

A study by Yuanyuan (2024) also confirmed the inhibitory impact of exercise on necroptosis in a type 2 diabetes mellitus (T2DM) mouse model. After 7 weeks of high-intensity interval training (HIIT), lactate dehydrogenase release, Necroptosis-related apoptosis proteins, and NLRP3 inflammasome expression were significantly reduced in the hippocampus. This indicates that exercise not only suppresses the RIPK1-RIPK3-MLKL pathway but also reduces neuroinflammation by modulating the NLRP3 inflammasome.

In conclusion, exercise intervention shows therapeutic potential by modulating the RIPK1-RIPK3-MLKL pathway, suppressing necroptosis, and mitigating inflammation. Exercise effectively inhibits the TNF-*α*/TNFR1 signaling pathway, reducing the necroptosis cascade, oxidative stress, and inflammatory factor release, ultimately protecting hippocampal neurons and improving cognitive function.

#### 5.2.3 Potential mechanism of necroptosis regulated by autophagy-lysosome for the treatment of Alzheimer’s disease

The autophagy-lysosome system plays a critical role in the pathogenesis of AD, and its dysfunction contributes substantially to the accumulation of Aβ and Tau proteins, while also triggering necroptotic apoptotic pathways that exacerbate neuronal damage (Zhao et al., 2018). Exercise, as a potent physiological regulator, has demonstrated significant potential in ameliorating these processes by enhancing autophagy-lysosomal function through multiple pathways, offering a promising therapeutic avenue for AD. Specifically, exercise has been shown to activate AMPK and PI3K/Akt signaling pathways, thereby modulating autophagy-lysosomal activity and suppressing necroptosis, suggesting novel therapeutic strategies for AD (Pena et al., 2020).

The AMPK signaling pathway serves as a key regulatory hub for exercise-induced autophagy. Activation of AMPK by exercise suppresses mTOR, promotes autophagosome formation, and restores lysosomal function, thereby improving cellular clearance of Aβ (Zhao et al., 2018). In a study involving 3-month-old APP/PS1 transgenic mice, 20 weeks of running training significantly activated the AMPK/mTOR pathway, promoted autophagosome-lysosome fusion, enhanced autophagic flux, reduced Aβ aggregation, inhibited RIPK1/RIPK3/MLKL signaling, and ultimately reduced necroptosis (Zhao et al., 2018). Furthermore, activation of the PI3K/Akt pathway by exercise enhances autophagy-lysosome regulation by promoting cell survival and inhibiting apoptosis. In a 3 × Tg-AD mouse model, a 9-week aerobic exercise regimen elevated insulin-like growth factor 1 (IGF-1) levels, activated the PI3K/Akt pathway, improved autophagy-lysosome function, reduced Aβ accumulation, and suppressed necroptosis, ultimately improving cognitive function (Pena et al., 2020).

Mitochondrial dysfunction is a key feature of neuronal damage in AD, with impaired mitochondria causing energy production deficits and releasing reactive oxygen species (ROS) that induce oxidative stress and activate RIPK1/RIPK3/MLKL signaling, triggering necroptosis and worsening neuronal injury (Fu et al., 2021). Mitophagy—a crucial mechanism for mitochondrial quality control—is essential for removing damaged mitochondria and maintaining mitochondrial homeostasis. Exercise markedly enhances mitophagy by activating autophagy-related pathways, thereby reducing ROS production, inhibiting necroptosis, and mitigating neuronal injury (Fu et al., 2021). In a murine model of calcium chloride and acetylcholine (CaCl2-Ach)-induced atrial fibrillation, three weeks of swimming training reduced protein levels of RIP1, RIP3, and their phosphorylated forms, as well as decreased MLKL translocation to the cell membrane. Exercise-induced AMPK/mTOR-mediated autophagy signaling reduced necroptosis (Fu et al., 2021). Although this study involved atrial fibrillation, the autophagy-necroptosis regulatory mechanism may also be relevant in AD.

Leem et al. (2024) showed that in an MPTP-induced Parkinson’s disease model, four weeks of rotarod training significantly decreased MLKL expression and phosphorylation of RIPK1 and RIPK3, thereby inhibiting necroptotic signaling. Exercise also attenuates neuroinflammation by reducing microglial hyperactivation and pro-inflammatory molecule release (e.g., iNOS, IL-1β, TNF-α), thereby inhibiting the NF-κB pathway. In addition, exercise upregulated antioxidant enzymes (MnSOD, NQO1, HO-1) and pathways (AMPK/Nrf2, SIRT3/FoxO3a), providing substantial neuroprotection against oxidative stress (Yang et al., 2019; Gureev et al., 2020; Fu et al., 2021).

The AMPK/Nrf2 and SIRT3/FoxO3a pathways interact within the autophagy-lysosome system to maintain cellular homeostasis, energy balance, and resilience against oxidative stress. In APP/PS1 transgenic mice, prolonged running training enhanced mitochondrial autophagy, decreased RIPK1 and pMLKL expression, reduced ROS accumulation, and ultimately inhibited RIPK1/RIPK3/MLKL signaling, safeguarding neurons from necroptotic damage (Liu et al., 2013). This provides compelling evidence that exercise can influence AD progression by modulating mitochondrial autophagy.

Recently, Transcription Factor EB (TFEB), a master regulator of autophagy and lysosomal function, has garnered significant attention in AD research (Iyaswamy et al., 2022). TFEB regulates autophagy-lysosome gene expression, promotes autophagosome formation, and supports lysosome biosynthesis, enhancing the cellular capacity to clear harmful proteins such as Aβ and Tau. Exercise can stimulate TFEB translocation to the nucleus, increasing autophagy and lysosomal gene expression, boosting the clearance of pathological proteins, and inhibiting necroptosis by blocking RIPK1/RIPK3/MLKL activation (Iyaswamy et al., 2022). In APP/PS1 mice, prolonged running enhanced nuclear TFEB translocation, restored lysosomal function, reduced Aβ and Tau levels, inhibited RIPK1/RIPK3 activation, and improved cognitive outcomes (Li et al., 2024).

In summary, exercise regulates autophagy-lysosome function by activating pathways such as AMPK, PI3K/Akt, and TFEB, thereby promoting the clearance of pathogenic proteins, reducing neuronal damage, and mitigating necroptosis through inhibition of the RIPK1/RIPK3/MLKL pathway.

#### 5.2.4 Mechanism of the role of exercise-regulated exosomal miR-215-5p expression in necroptosis in Alzheimer’s disease

Recent studies indicate that exercise intervention holds considerable promise in preventing AD, particularly regarding controlling cellular survival and apoptosis through the modulation of exosomal miRNAs. Exosomes are membrane-bound vesicles secreted by cells that transport physiologically active substances such as miRNAs, proteins, and DNA and are involved in intercellular signaling. Exosomes penetrate the central nervous system across the blood–brain barrier and contribute to the degenerative mechanisms of AD (Chen et al., 2022). Exercise can markedly enhance miR-215-5p levels in exosomes, subsequently influencing the expression of numerous genes and proteins associated with necroptosis, particularly isocitrate dehydrogenase 1, SIRT1, and Bcl-2-like protein 11. These compounds are integral to the necroptosis signaling pathway (Chen et al., 2022).

IDH1 is a crucial metabolic regulatory enzyme that significantly influences cellular energy metabolism and the oxidative stress response. IDH1 influences intracellular energy metabolism by modulating the intermediates of the citric acid cycle. Research indicates that exercise-induced upregulation of miR-215-5p mitigates oxidative stress damage to neurons by targeting and suppressing IDH1 expression, therefore decreasing necroptosis incidence. SIRT1 is a crucial deacetylase that regulates multiple metabolic pathways and inflammatory responses. SIRT1 can deacetylate many transcription factors, including FOXO and p53, thereby suppressing apoptosis and inflammatory responses. BCL2L11, a pivotal member of the Bcl-2 family, is directly implicated in the control of the apoptotic pathway. Exercise-induced miR-215-5p can directly target and suppress the expression of BCL2L11, hence inhibiting its pro-apoptotic action and lowering neuronal mortality. This process promotes neuronal survival by diminishing Caspase-3 cleavage and obstructing the activation of apoptotic signals. Furthermore, exercise can modulate the RIPK1-RIPK3-MLKL necroptotic apoptotic pathway by regulating exosomal miRNAs. TNF-*α* interacts with its receptor TNFR1 to activate RIPK1, phosphorylating RIPK3. RIPK3 subsequently activates MLKL, which relocates to the cell membrane, resulting in membrane rupture and initiating cell Necroptosis. Exercise markedly suppresses the phosphorylation of RIPK1 and RIPK3 by enhancing the expression of miR-215-5p, which diminishes the activation of MLKL, averting cell membrane rupture and decreasing the incidence of necroptosis. The research demonstrated that exercise can enhance the expression of BDNF, which is linked to neuroprotection, via modulating exosomal miR-215-5p. BDNF stimulates the PI3K-Akt and MAPK-Erk1/2 signaling pathways, enhancing neuronal survival, synaptic plasticity, and nerve regeneration. The upregulation of BDNF boosts neuronal anti-apoptotic capacity, improves cognitive function, and decelerates the degenerative progression of AD by repairing damaged synapses. Exercise enhances BDNF expression by competitively suppressing miR-382-3p, improving neuronal repair capacity. Simultaneously, exercise markedly diminishes the inflammatory response and oxidative stress in the brains of AD patients via upregulating exosomal miR-215-5p. Research indicates that exercise can suppress the chronic inflammatory response induced by necroptosis by modulating exosomal miRNAs to diminish the secretion of pro-inflammatory cytokines such as IL-6 and TNF-α. The upregulation of exosomal miR-215-5p diminishes the inflammatory response by modulating molecules like SIRT1 and IDH1 while simultaneously enhancing the clearance of cytotoxic substances through the activation of the autophagy-lysosome system, thus mitigating the pathological damage associated with AD.

Wang et al. (2023a) investigated the influence of aerobic exercise on type 2 diabetic mice by modulating the MALAT1/miR-382-3p/BDNF signaling pathway through a series of experiments, mainly focusing on the molecular mechanism by which exercise enhances cognitive performance via serum exosomes. The experimental methodology involved the creation of a T2DM mouse model, the induction of diabetes using a high-fat diet coupled with streptozotocin, and implementing a 12-week treadmill aerobic exercise intervention. The research identified differentially expressed lncRNAs and mRNAs using transcriptome sequencing of serum exosomes and confirmed the target gene interaction between MALAT1 and miR-382-3p. Subsequently, the impact of aerobic exercise on the survival, proliferation, apoptosis, and insulin resistance of hippocampal neurons in mice was assessed both *in vitro* and *in vivo*. The findings indicated that the expression level of MALAT1 in the serum exosomes of T2DM mice was decreased, but the expression of miR-382-3p was elevated. Aerobic exercise markedly elevated MALAT1 expression and reduced miR-382-3p levels. MALAT1 enhanced BDNF expression by suppressing miR-382-3p, thereby ameliorating mouse cognitive deficits. *In vitro*, research has shown that the silencing of MALAT1 or the overexpression of miR-382-3p diminished the expression of INSR, IRS-1, and the PI3K/AKT signaling pathways, suppressed neuronal growth, and enhanced death. In the aerobic exercise intervention group, the expression of MALAT1 in mouse serum was markedly elevated, the level of miR-382-3p was dramatically diminished, and both glucose tolerance and insulin resistance exhibited improvement. The findings from the Morris water maze experiment indicated that the escape latency of the exercise group mice was markedly reduced, while the duration spent in the target quadrant was prolonged, implying an enhancement in their cognitive function. The experiment concludes that aerobic exercise enhances cognitive function in type 2 diabetic mice by modulating the MALAT1/miR-382-3p/BDNF signaling pathway, facilitating neuronal growth and suppressing death. Kordi et al. (2024) investigated the potential function of exercise in panapoptosis and cardiovascular disease, concluding that exercise can enhance cardiovascular health by upregulating exosome expression.

#### 5.2.5 Mechanism of O-GlcNAc glycosylation in the regulation of movement and its role in necroptosis in Alzheimer’s disease

Glycosylation, a prevalent post-translational modification in eukaryotic cells, entails the covalent linkage of oligosaccharides to designated amino acid residues of proteins through glycosidic bonds. This alteration pathway governs protein folding, processing, and function, significantly impacting learning and memory (Du et al., 2021). O-glycosylation and N-glycosylation are the two most prevalent forms of glycosylation. N-glycosylation is a multifaceted process in the endoplasmic reticulum and Golgi apparatus, regulated by many glycosidases and glycosyltransferases. The Tau protein, primarily in the cytoplasm, typically does not experience N-glycosylation modification. Conversely, O-GlcNAc glycosylation selectively modifies serine and threonine residues, and its mechanism resembles that of phosphorylation; specifically, a single N-acetylglucosamine (O-GlcNAc) is attached to the hydroxyl group of the Ser or Thr side chain of the target protein by an O-glycosidic bond. O-GlcNAc glycosylation, a specific instance of O-glycosylation, is crucial in cell signaling, gene expression regulation, and cellular response. O-GlcNAc glycosylation predominantly occurs on proteins within the nucleus and cytoplasm, allowing for the modification of Tau protein at specific serine and threonine residues. Research indicates that the O-GlcNAc glycosylation levels of Tau protein in the brains of AD patients and model mice are markedly lower than those in the control group (Gatta et al., 2016). Elevating the O-GlcNAc glycosylation of Tau protein via pharmacological intervention can mitigate AD symptoms (Diwu et al., 2013). However, suppressing this glycosylation process may worsen the condition (Lim et al., 2015). This indicates that augmenting the O-GlcNAc glycosylation of Tau protein could be viable for treating AD. Subsequent research has demonstrated that the O-GlcNAc glycosylation sites on Tau protein coincide with phosphorylation sites, including Ser396/404 and Thr205, and these modifications are mutually exclusive at the exact location: a reduction in O-GlcNAc glycosylation results in excessive phosphorylation of Tau protein, thereby facilitating its aggregation; conversely, it can suppress phosphorylation (Liu et al., 2004). Moreover, the competitive interaction between the two is substantiated by the observation that enhancing Tau protein phosphorylation with a phosphatase inhibitor and okadaic acid results in a commensurate decrease in the O-GlcNAc glycosylation level of Tau protein (Lefebvre et al., 1999). Consequently, augmenting the O-GlcNAc glycosylation of Tau protein by inhibiting its excessive phosphorylation may mitigate the degenerative progression of AD by establishing a positive feedback loop that suppresses phosphorylation. Research indicates that O-GlcNAc glycosylation can inhibit necroptosis, providing a protective benefit for AD. In AD models, augmented necroptosis has been seen; however, in the 5xFAD mouse model, RIPK3 O-GlcNAc decreased its activity and decelerated the advancement of necroptosis. Augmented O-GlcNAc glycosylation alleviates AD symptoms, diminishes Aβ accumulation and neuroinflammation, rehabilitates mitochondrial function, and reinstates normal microglial activity (Park et al., 2021). This indicates that O-GlcNAc glycosylation not only serves a protective function in advancing AD but also decelerates the illness’s progression by modulating cell survival mechanisms, including inhibiting necroptosis (Ye et al., 2023).

Recent studies indicate that physical activity may mitigate the pathological alterations associated with AD by modulating the phosphorylation of Tau protein, particularly at the Ser396/404 location (Liu et al., 2013), while O-GlcNAc glycosylation has a negative regulatory effect on Tau protein phosphorylation. Exercise may reduce necroptosis in AD via modulating O-GlcNAc glycosylation levels in conjunction with the effects of O-GlcNAc glycosylation on necroptosis. Prior research indicates that diminished O-GlcNAc glycosylation of Tau protein facilitates its hyperphosphorylation, resulting in Tau protein aggregation and neuronal impairment, with these alterations being closely associated with heightened necroptosis in AD. Necroptosis significantly contributes to the pathological progression of AD, and increased O-GlcNAc glycosylation can impede these cell death mechanisms. This indicates that O-GlcNAc glycosylation may mitigate AD pathology by inhibiting Tau protein phosphorylation and preventing neuronal damage caused by necroptosis. Moreover, exercise enhances O-GlcNAc glycosylation levels by upregulating the activity of O-GlcNAc transferase (OGT). Treadmill exercise enhances OGT activity in the cardiac muscle of diabetic mice (Cox and Marsh, 2013). This indicates that OGT and O-GlcNAcase are responsive to exercise, which enhances O-GlcNAc glycosylation through increased OGT activity, perhaps inhibiting hyperphosphorylation and necroptosis by modulating Tau protein O-GlcNAc glycosylation.

**Figure 6:**
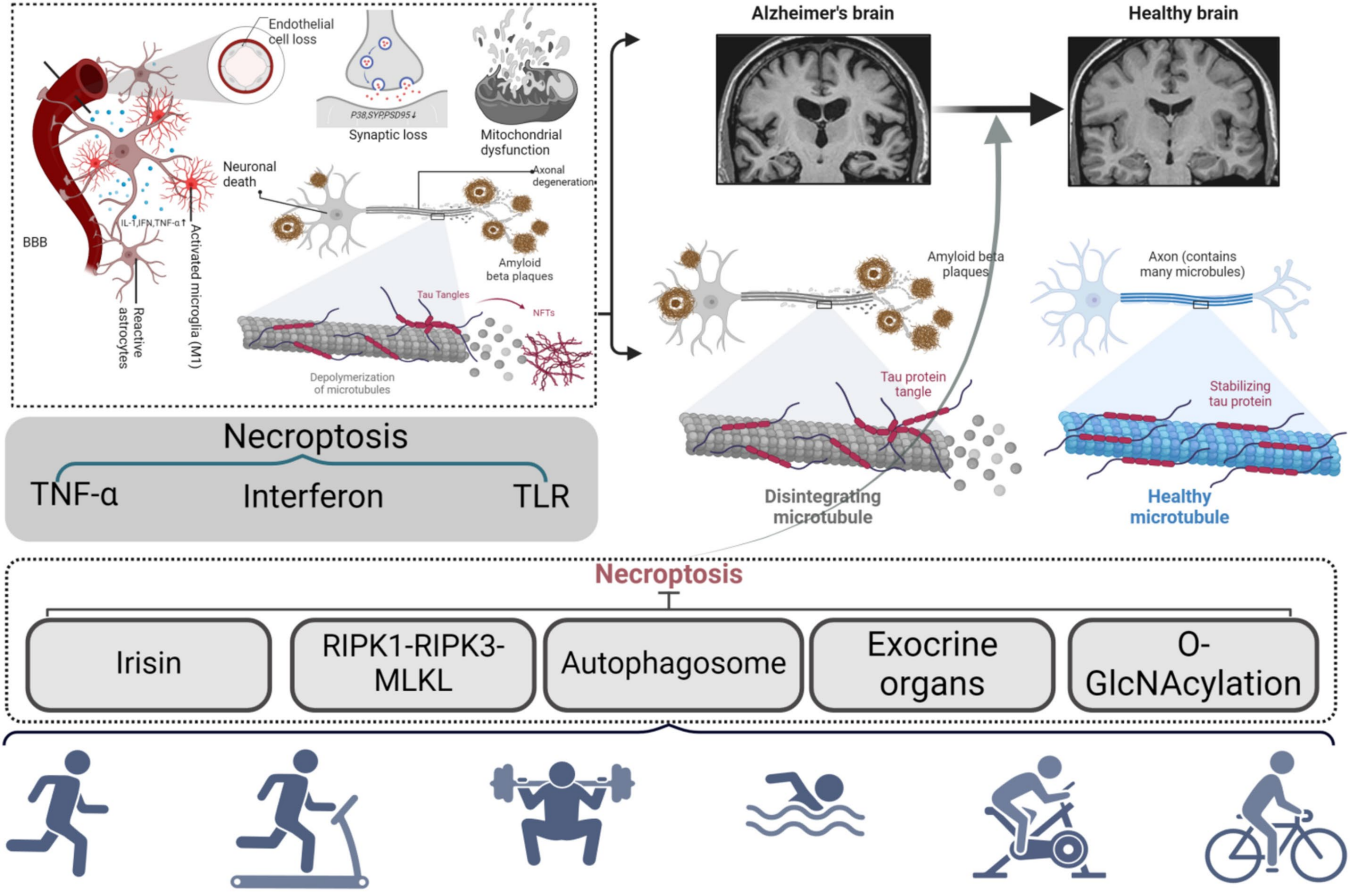
Potential mechanism of necroptosis-mediated AD motion prevention.

While current studies indicate that exercise can modulate O-GlcNAc glycosylation and indirectly influence Tau protein phosphorylation, thereby safeguarding neurons from necroptosis, there is a paucity of research directly demonstrating that exercise mitigates necroptosis in AD by regulating the activity of OGT and OGA in the brain. Consequently, while this indirect evidence suggests that exercise may impede necroptosis through the modulation of O-GlcNAc glycosylation, additional research is required to elucidate the precise mechanism by which exercise exerts this inhibitory effect on necroptosis in AD, as seen in (Figure 6).

### 5.3 What should future efforts focus on?

#### 5.3.1 Exercise’s protective effects on Alzheimer’s: from mice to humans

Exercise interventions have been widely explored for their potential to delay brain aging and ameliorate neurodegenerative diseases. However, translating findings from rodent models into human applications poses significant challenges. Evidence from rodent studies demonstrates that exercise can attenuate AD pathology by modulating neurotrophic factors—such as BDNF—and autophagy pathways, suppressing necroptosis, reducing Aβ accumulation, and inhibiting tau hyperphosphorylation, thereby enhancing cognitive function (Heyn et al., 2004; Weuve et al., 2004). Nonetheless, these mechanisms have not been consistently reproduced in humans, likely due to substantial differences between rodents and humans in brain structure, metabolic state, and blood–brain barrier permeability. Addressing these translational gaps requires identifying the key discrepancies in signaling pathways and pathological features and validating these mechanisms in human clinical trials (De la Rosa et al., 2020).

Moreover, the heterogeneity in genetic background and disease status among individuals can further influence the efficacy of exercise interventions. For example, APOE ε4 carriers may exhibit reduced responsiveness to exercise-induced neuroprotection, necessitating combination with other therapeutic strategies to optimize outcomes (de Frutos Lucas et al., 2023). Therefore, future research should adopt a precision medicine approach, leveraging multi-omics analyses—including genomics and metabolomics—to design tailored exercise interventions for different subpopulations and validate their effectiveness in large-scale clinical studies. Such endeavors will facilitate the precision application of exercise in the prevention and treatment of brain aging and AD, ultimately bridging the gap from basic research to clinical practice.

#### 5.3.2 Impact of individual differences on exercise interventions in AD

Different stages of AD require distinct treatment objectives and tailored exercise interventions, marking an essential step toward precision medicine for AD (Sagud et al., 2021). For individuals with mild cognitive impairment (MCI), the primary aim should be to reduce progression to AD and decelerate its rate. Evidence indicates that exercise therapy at this stage ranks only second to music therapy in enhancing cognitive function among MCI patients (Lai et al., 2020). Moreover, when combined with pharmacological treatments such as acetylcholinesterase inhibitors, exercise has shown remarkable effects. Thus, a multimodal intervention incorporating combined exercise regimens, music therapy, and conventional medications may enhance neuroplasticity in early-stage MCI patients. Additionally, incorporating psychoeducation into this comprehensive strategy may bolster patients’ confidence in the treatment process (Cocchiara et al., 2020). For those in the mild to moderate stages of AD, the goal is to slow disease progression. Physical exercise, as part of an integrated approach involving cognitive and music therapy, has been shown to support neuroplasticity in this patient group. It can also lead to modest improvements in cognitive function and alter patterns of resting brain activity (Sagud et al., 2021). In the advanced stages of AD, interventions like walking, limb movements, and clapping have demonstrated benefits primarily for mood enhancement and psychological well-being, though their impact on halting disease progression appears limited (Sagud et al., 2021).

**Figure 7:**
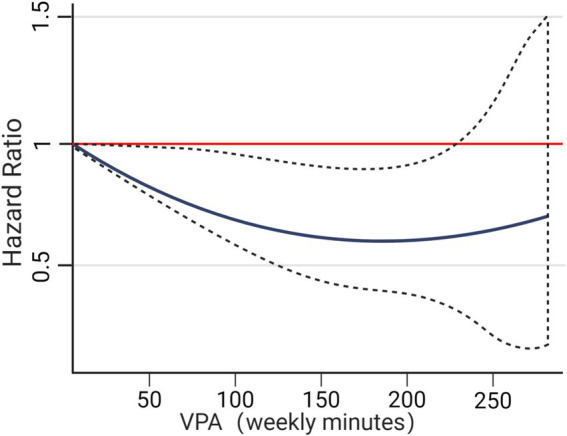
Dose-response relationship between VPA and Alzheimer’s disease.

Different forms of exercise yield varying effects on cognitive function in AD patients (Heisz and Waddington, 2023). A meta-analysis by Susana et al. (López-Ortiz et al., 2021) demonstrated that aerobic exercise significantly enhances cognition in AD patients, while the network meta-analysis by Shi et al. found resistance training to be the most effective. Daniel et al.’s (Gallardo-Gomez et al., 2022) dose–response meta-analysis established a nonlinear relationship between exercise dose and cognitive function in the elderly, revealing that even doses below the WHO recommendation (724 MET-min/week) can yield cognitive benefits. Yuan et al. (2024) conducted a dose–response meta-analysis exploring the unique effects of different exercise doses on cognitive outcomes in AD patients. Their findings confirmed a nonlinear relationship, with significant improvement seen at doses up to 1,000 MET-min/week, but diminished effects beyond this level. The optimal overall dose for enhancing cognitive function was identified as 650 MET-min/week, equivalent to 150 min of moderate or 75 min of high-intensity exercise per week. Specifically, 660 MET-min/week was most effective for aerobic exercise (Figure 7). Notably, mixed exercise showed efficacy at lower doses (180 MET-min/week), while resistance training and Tai Chi did not demonstrate a significant dose–response relationship. The identified optimal dose aligns with WHO’s recommended range of 600–1,200 MET-min/week, affirming the value of these guidelines. Considering these findings, along with individual variability in exercise response among the elderly and the ACSM physical activity guidelines (Garber et al., 2011), we propose an exercise prescription for AD patients based on the FITT framework (Heisz and Waddington, 2023):

1. Frequency: Engage in exercise as frequently as possible.
2. Intensity: Moderate to high intensity.
3. Time:
  Adhere to long-term, regular exercise.
  Any exercise is better than none.
  Weekly dose to achieve cognitive improvement:
  Minimum: 70 min of moderate intensity or 35 min of high intensity.
  Optimal: 140 min of moderate intensity or 75 min of high intensity.
4. Type: Aerobic (e.g., walking, swimming, cycling), resistance training (using body weight, bands, or equipment), multi-component exercise (balance, resistance, and aerobic), Tai Chi or yoga.

#### 5.3.3 What research should be conducted in the future?

##### 5.3.3.1 Limitations of the current study

Despite growing interest in the impact of exercise on AD pathology, substantial research gaps persist, particularly in elucidating how exercise regulates necroptosis mechanisms. Chen et al. (2022) were the first to propose that exercise might suppress necroptosis by modulating exosomal miRNAs, such as miR-215-5p, thereby mitigating neuronal damage. Their study demonstrated that exercise reduced activation of the RIPK1-RIPK3-MLKL signaling cascade in AD models and ameliorated neuroinflammation associated with necroptosis by modulating IDH1, BCL2L11, and SIRT1 expression. However, the study’s limitations include its reliance on murine models without validation in human clinical trials, leaving the precise role of exosomal miRNAs and their specific regulatory mechanisms in AD unaddressed.

Similarly, Zeini Zadeh et al. (2023) investigated the effects of complex voluntary exercise on hippocampal necroptosis-related gene expression in an AD rat model. The study revealed a significant reduction in hippocampal RIPK1 and MLKL levels, accompanied by partial improvement in spatial memory function. However, although the intervention diminished necroptosis, it did not completely restore cognitive function, suggesting that necroptosis may interact with other cell death modalities, such as apoptosis and ferroptosis. This interaction indicates that a single exercise modality may be insufficient to fully halt AD pathology progression. Likewise, Yuanyuan (2024) reported that high-intensity interval training (HIIT) attenuated necroptosis markers, including RIPK1, RIPK3, and MLKL, in diabetic mouse models while also reducing pro-inflammatory cytokine release (e.g., TNF-*α*, IL-6, IL-1β). Nevertheless, these findings are limited by the differences in pathogenesis between diabetes and AD, constraining the translatability of results.

Further indirect evidence supports the role of exercise in regulating necroptosis. For instance, Fu et al. (2021) demonstrated that aerobic exercise reduced myocardial fibrosis and inflammatory responses via inhibition of the RIPK1-RIPK3-MLKL axis in atrial fibrillation models, thus improving cardiac function. Although this suggests a role for necroptosis in cardiovascular diseases, its direct relevance to AD remains unclear. Ghardashi Afousi et al. (2019) showed that HIIT ameliorated myocardial ischemia/reperfusion injury by downregulating RIPK3 and MLKL expression, providing a theoretical framework for exploring HIIT in AD contexts. However, these studies lack direct evidence for AD, limiting their applicability. Additionally, Leem et al. (2024) observed that exercise combined with creatine supplementation reduced α-synuclein aggregation and necroptosis in Parkinson’s disease (PD) models by downregulating RIPK1 and MLKL. Yet, due to the distinct pathophysiological mechanisms of PD and AD, the relevance of these findings to AD remains speculative.

Ethical considerations or challenges: Drawing on established guidelines such as those outlined by Harriss et al. (2022) on ethical standards in sports and exercise science, we propose the following additions:

1. Informed consent and cognitive impairment: Ensuring informed consent in individuals with cognitive decline is challenging but essential. Ethical research must involve appropriate surrogate decision-makers or guardians and prioritize clear, accessible communication tailored to the participants’ comprehension levels.
2. Balancing benefits and risks: While exercise may provide cognitive and physical benefits, risks such as falls or cardiovascular events should be carefully assessed. Pre-study risk evaluations and individualized exercise prescriptions are necessary to minimize harm.
3. Equity and accessibility: Ethical considerations must include equitable access to interventions, especially for socioeconomically disadvantaged populations or those in care facilities, where resources may be limited.
4. Caregiver burden: Ethical frameworks should address the additional responsibilities placed on caregivers, ensuring interventions are feasible and supportive of the caregiving dynamic.
5. Data privacy and remote methods: For studies involving remote monitoring or digital platforms, adherence to stringent data protection and privacy standards is critical, as outlined in the use of electronic consent and secure data handling protocols.

##### 5.3.3.2 Future directions

**Figure 8:**
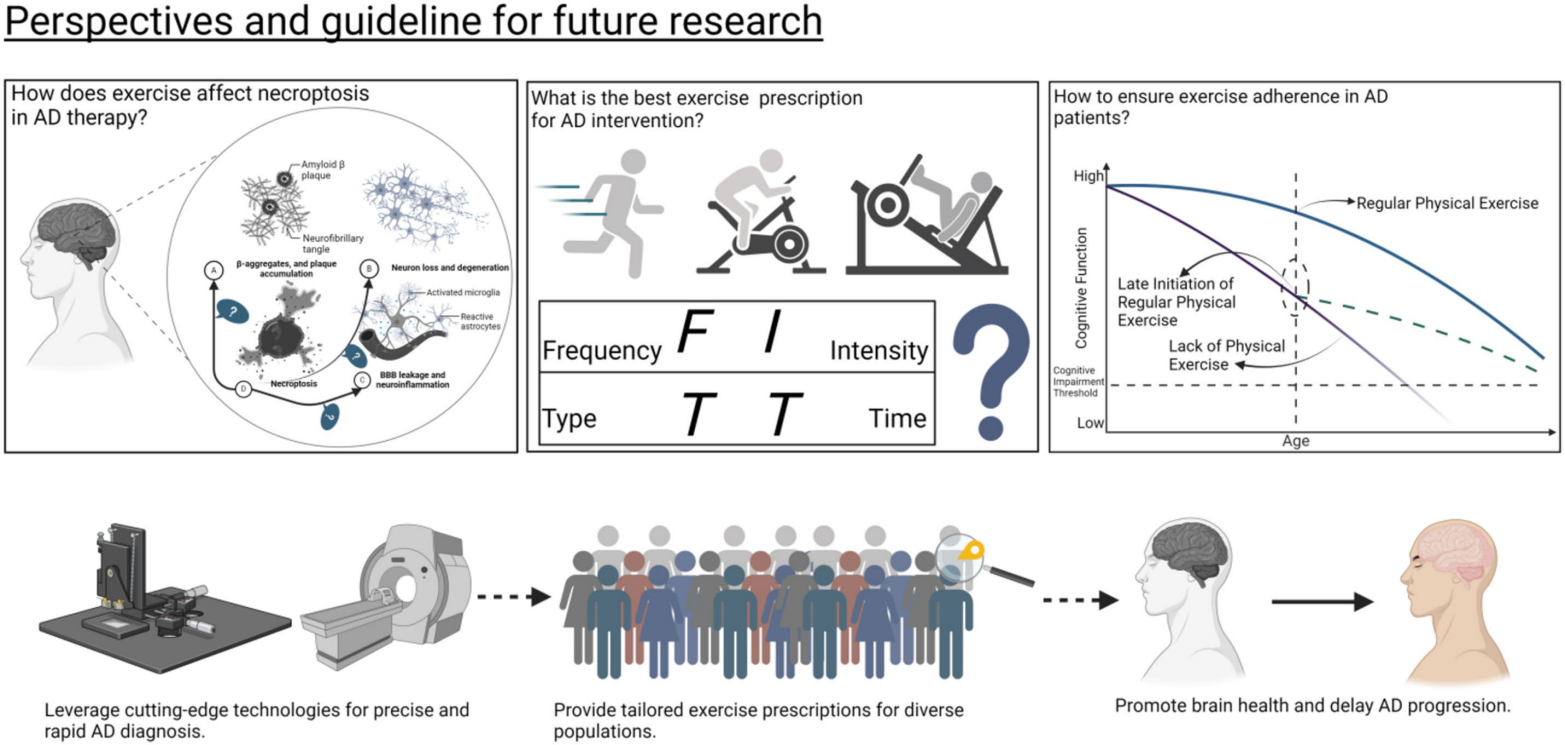
Summary of future research directions on exercise and AD.

Future research should focus on large-scale, multicenter randomized controlled trials (RCTs) to systematically evaluate the long-term effects of different exercise modalities on necroptosis in Alzheimer’s disease (AD) and their links to cognitive improvements. Standardized intervention protocols—including intensity, frequency, and duration—must account for individual variability such as genetic predisposition, disease stage, and overall health. Combining exercise with existing therapies, investigating how exercise interacts with other cell death mechanisms, such as apoptosis and ferroptosis, will be pivotal. Multi-omics approaches, including single-cell RNA sequencing, proteomics, and metabolomics, could uncover critical nodes in necroptosis regulation and illuminate molecular networks underpinning exercise’s neuroprotective effects. The interplay between necroptosis and neuroinflammation should also be examined, particularly how exercise modulates microglial activation and reduces pro-inflammatory cytok developing necroptosis-related biomarkers (e.g., RIPK1, RIPK3, MLKL, TNF-*α*, IL-6) is essential for early monitoring and intervention efficacy evaluation. Neuroimaging tools like MRI and PET could provide insights into structural and functional brain changes associated with exercise, offering evidence of delayed cognitive decline and disease progression. By integrating these findings through interdisciplinary collaboration, personalized exercise strategies may emerge, laying a robust foundation for AD prevention and treatment (see Figure 8).

## 6 Conclusion

Necroptosis, through the RIPK1-RIPK3-MLKL pathway, plays a critical role in AD pathogenesis, contributing to neuronal death and cognitive decline. Exercise interventions may modulate this pathway, promoting neuronal survival and reducing inflammation. However, the precise molecular mechanisms remain insufficiently understood. Future research should explore how different exercise types and intensities influence necroptosis and apoptosis in AD, particularly their effects on cognitive function, neurodegeneration, and disease progression. Investigating the interaction between exercise and neuroinflammation, oxidative stress, and mitochondrial dysfunction will be crucial. Clinically, such insights could lead to tailored exercise-based interventions that not only complement existing pharmacological therapies but also provide a viable non-pharmacological treatment option for AD, potentially modifying disease progression and improving patient outcomes over the long term.
